# Heart rate, intelligence in adolescence, and Parkinson’s disease later in life

**DOI:** 10.1007/s10654-021-00730-y

**Published:** 2021-03-06

**Authors:** Elisa Longinetti, Yiqiang Zhan, Mizuki Sata, Henrik Larsson, Brian M. D′Onofrio, Hiroyasu Iso, Karin Wirdefeldt, Fang Fang

**Affiliations:** 1 Department of Medical Epidemiology and Biostatistics, Karolinska Institutet, Stockholm, Sweden; 2 Institute of Environmental Medicine, Karolinska Institutet, Box 210, 171 77 Stockholm, Sweden; 3 German Center for Neurodegenerative Diseases, Ulm, Germany; 4 Department of Preventive Medicine and Public Health, Keio University School of Medicine, Tokyo, Japan; 5 Public Health, Department of Social Medicine, Osaka University Graduate School of Medicine, Suita, Japan; 6 Department of Public Health Medicine, Faculty of Medicine, and Health Services Research and Development Center, University of Tsukuba, Tsukuba, Japan; 7 Faculty of Medical Sciences, Örebro University, Örebro, Sweden; 8 Department of Psychological and Brain Sciences, Indiana University, Bloomington, USA; 9 Department of Clinical Neuroscience, Karolinska Institutet, Solna, Sweden

**Keywords:** Parkinson’s disease, Epidemiology, Risk factor, Early-life exposure, Cohort study

## Abstract

To investigate whether physical and cognitive fitness measured in late adolescence was associated with future risk of Parkinson’s disease (PD). The cohort included 1,259,485 Swedish men with physical fitness, body mass index (BMI), resting heart rate (RHR), blood pressure, intelligence quotient (IQ), and stress resilience measured at the age of 17–20 in relation to conscription. Incident cases of PD were ascertained from the Swedish Patient Register. Hazard ratios were estimated from Cox models, after controlling for multiple confounders. We further performed Mendelian randomization (MR) analyses to assess the causality of the associations, using GWAS summary statistics with > 800,000 individuals. During follow-up, we identified 1,034 cases of PD (mean age at diagnosis = 53). Men with an RHR > 100 beats per minute had a higher risk of PD compared to men with an RHR of 60–100 beats per minute (HR = 1.47; 95% CI = 1.08–1.99). Men with IQ above the highest tertile had a higher risk of PD compared to men with an IQ below the lowest tertile (HR = 1.46; 95% CI = 1.19–1.79). We found no association for physical fitness, BMI, blood pressure, or stress resilience. A causal relationship was suggested by the MR analysis between IQ and PD, but not between RHR and PD. RHR and IQ in late adolescence were associated with a higher risk of PD diagnosed at relatively young age. The association of IQ with PD is likely causal, whereas the association of RHR with PD suggests that altered cardiac autonomic function might start before 20 years of age in PD.

**Supplementary Information:**

The online version contains supplementary material available at 10.1007/s10654-021-00730-y.

## Introduction

Environmental, psychosocial, and behavioural factors are likely all involved in the development of Parkinson’s disease (PD) [1]. Physical activity is a lifestyle factor associated with a reduced risk of PD [2]. A potential protective role of physical fitness on PD has also been suggested [3]. Other correlates of physical activity include body composition and cardiorespiratory endurance [4]. The role of body mass index (BMI) on PD has been studied with inconclusive results [5], although Mendelian randomization (MR) analysis supports an inverse association between BMI and PD [6]. Cardiac function impairment might represent the earliest stage of PD [7], but results are inconsistent across studies [8–11]. Cognitive and emotional factors are also important correlates of physical activity [4]. However, no study has examined the role of intelligence quotient (IQ) or stress resilience on PD risk.

PD has a prodromal stage spanning over decades [12]. Assessing causal relationship between a risk factor and PD is therefore not always straightforward in observational studies, even when the risk factor does occur before the clinical onset of PD. MR analysis has the potential to investigate causal relationship between a risk factor and a disease, avoiding common methodological limitations of observational studies including reverse causation [13]. To this end, we examined the associations of physical fitness, BMI, resting heart rate, blood pressure, IQ, and stress resilience measured in late adolescence with PD in a cohort of more than one million Swedish men. We further performed a MR analysis, using genome-wide association study (GWAS) summary statistics from > 800,000 individuals. We hypothesize that corroborating findings from both analyses would more likely suggest causation.

## Methods

### Cohort analysis

#### Study design

Since 1968 the Swedish Conscript Register has been collecting data on all Swedish men that underwent conscription examinations. Attending conscription examination was mandatory in Sweden for all men around 18 years of age, except those with severe physical or mental disabilities. During the examination, trained healthcare professionals collected detailed data on health status, including physical fitness, BMI, resting heart rate (RHR), blood pressure, IQ, and stress resilience. Details about these measurements have been described elsewhere [14]. In brief, physical fitness was measured by the maximum working capacity in Watts that an individual could sustain on a progressively increasingly loaded electric bicycle until exhaustion, for a maximum of six minutes. BMI was calculated from weight and height whereas RHR was measured after 5–10 min of rest in the supine position. Blood pressure was also measured after at least five minutes of rest in the supine position, using sphygmomanometers placed in the right upper arm. A total IQ score was obtained by summing scores of written tests with progressive difficulty investigating verbal IQ, spatial and technical ability, and general knowledge. A clinical psychologist evaluated psychological functioning, a proxy for stress resilience, during a 20–25 min semi-structured interview that investigated different psychological dimensions. IQ and stress resilience values were transformed to a standard 9-level stanine scale.

In the present study, we included in the cohort all 1, 232, 364 Swedish men that participated in the conscription examination at age 17–20 during 1968–1993. Using the unique personal identification numbers assigned to all Swedish residents [15], we individually followed these conscripts from their 40th birthday to the date of PD diagnosis, emigration out of Sweden, death, or end of follow-up (December 31st, 2013), whichever occurred first, through cross-linkages to the Swedish Patient Register [16], the Causes of Death Register [17], the Migration Register [18], the Swedish Multi-Generation Register [19], and the Swedish Population and Housing Censuses. We started the follow-up from 40 years of age because PD cases diagnosed at very young age are more likely of genetic causes compared to PD cases diagnosed at later ages. The Swedish Patient Register collects hospital discharge diagnoses since 1964 (with nationwide coverage since 1987) and hospital-based specialist outpatient care since 2001 (> 80% of the entire country). From the Swedish Patient Register, we identified conscripts that received a diagnosis of PD or Parkinsonian disorder during follow-up, according to the Swedish revisions of the International Classification of Disease codes (PD: ICD-7 350 between 1964 and 1968, ICD-8 342 between 1969 and 1986, ICD-9 332.0 between 1987 and 1996, and ICD-10 G20 from 1997 onward; Parkinsonian disorder: ICD-9 333.0 between 1987 and 1996, and ICD-10 F02.3, G21.4, G21.8, G21.9, G23.1, G23.2, G23.9, G25.9, G31.8 from 1997 onward). We defined the first date of hospital contact for PD as the date of PD diagnosis. The positive predictive value (PPV) of the PD definition based on inpatient discharge records of the Swedish Patient Register is satisfactory; 83% for main diagnosis and 71% for any diagnosis, compared to standard clinical evaluation [20]. Although a validation study is yet to be performed, the PPV of outpatient specialist-care based PD definition (2001 onward) is presumably greater. We identified the date of death from the Causes of Death Register and date of emigration out of Sweden from the Migration Register. We identified the parents of the conscripts from the Swedish Multi-Generation Register and linked the parents to the Swedish Population and Housing Censuses, conducted every five years by Statistics Sweden, to identify the parental socioeconomic status (SES) around the time of conscription examination.

We excluded from the analysis conscripts that were diagnosed with PD (N = 92), Parkinsonian disorders (N = 42), died (N = 21,405), or emigrated out of Sweden (N = 65,464) before the beginning of follow-up. We further excluded 42,310 conscripts with no information on physical fitness, BMI, RHR, blood pressure, IQ, or stress resilience, and 43,566 conscripts that were younger than 40 during the entire follow-up, leaving a total of 1,059,485 conscripts (85.9%) in the final analysis.

#### Statistical analysis

We used Spearman correlation coefficient (ρ) to test the correlations between physical fitness, BMI, RHR, blood pressure, IQ, and stress resilience. We used Cox models to estimate hazards ratios (HRs) and their corresponding 95% CIs for the risk of PD, comparing conscripts with different levels of the studied attributes. We used attained age as the underlying timescale in all analyses. We first fitted a simple model adjusted for calendar period of conscription examination (1968–1980 or 1981–1993) and parental socioeconomic status (blue collar, white collar, farmers, self-employed, or others). In order to evaluate the independent effect of the studied attributes, we fitted a full model where physical fitness, BMI, RHR, blood pressure, IQ, and stress resilience were adjusted for one another. As positive associations were noted for RHR, IQ, and PD risk, we further tested whether these associations were attributable to adult education and cardiovascular disease. We linked the study cohort to the Swedish Education Register, which collects information on the highest attained educational level of all individuals in Sweden at age 16 onward since 1985, and ascertained the highest educational level for all conscripts. Similarly, we linked the cohort to the Patient Register and identified any clinical diagnosis of cardiovascular disease after conscription but before end of follow-up, using ICD-7 codes 400–468, ICD-8 codes 390–458, ICD-9 codes 390–459, and ICD-10 codes I00-I99. In the statistical analysis, we adjusted the full Cox model with adult education and cardiovascular disease. To investigate if misdiagnosis might explain some of the associations, we conducted an additional analysis by restricting the definition of PD to conscripts who received a PD diagnosis as main diagnosis. Finally, we started the follow-up of the cohort from 40 years of age to exclude PD cases diagnosed at very young age, in another two sensitivity analyses we first started the follow-up from 50 years of age as another definition of early-onset PD and then performed an analysis without such age restriction.

### Mendelian randomization (MR) analysis

**Fig. 1:**
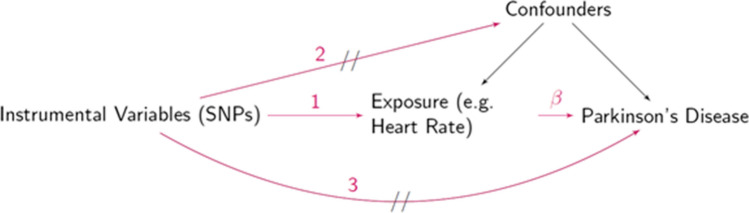
Mendelian randomization framework, In the MR framework, we are interested in testing and estimating the causal effects (β) of an exposure variable on the risk of Parkinson’s disease, in the presence of confounders including measured confounders and unmeasured residual confounding. The three conditions for MR analysis are: Instrumental variables are associated with the exposure of interest (e.g. arrow numbered 1 should exist).Instrumental variables are not associated with the confounders between the exposure and the outcome (e.g. arrow numbered 2 should not exist). Instrumental variables are not associated with the outcome when adjusting for the exposure and the potential confounders (e.g. there are no other pathways from instrumental variables to the outcome—arrow numbered 3 should not exist)

Because positive associations were noted between RHR, IQ, and PD, we further tested the causality of these associations in an MR framework (Fig. 1), using available GWAS summary statistics.

#### GWAS summary statistics for resting heart rate and intelligence

The GWAS for RHR was performed among 134,251 participants of the UK Biobank [21] The average age of these participants was 56.6 years, and 47.2% of the participants were male. We identified all genetic variants at 76 loci that are associated with RHR. RHR in the UK Biobank was assessed by two methods: an automated reading during blood pressure measurement and during arterial stiffness measurement using the pulse waveform obtained from the finger with an infrared sensor. Multiple measurements for one individual were averaged. Both genotyped and imputed variants were analysed with strict quality controls, leaving 19,941,912 variants for the analyses. A linear regression model was conducted assuming an additive genetic effect. Covariates included in the model were age, age-squared, sex, the first 10 principal components, and genotyping array.

The GWAS for intelligence was performed among 269,867 participants of 14 independent cohorts of European ancestry including 9,295,118 genetic variants that pass quality control [22]. Different measures of intelligence were assessed in each cohort but were all operationalized to index a common latent g factor underlying multiple dimensions of cognitive functioning. All cohorts extracted a summary score from a multidimensional set of cognitive performance tests and used this normally distributed score as the phenotype in a multivariable-adjusted (i.e., age, sex, and ancestry principal components) linear regression model, with the exception of the Health and Retirement Study, where a logistic regression model was performed with case status reflecting whether participants were drawn from an extreme-sampled population of very high intelligence versus a sample of unselected population controls. Stringent quality control measures were applied to the summary statistics for each GWAS cohort that was meta-analysed.

#### GWAS summary statistics for PD

The GWAS summary statistics for PD were obtained from the most recent public PD GWAS and 23andMe PD cohorts of European ancestry that were genotyped on custom arrays. The data comprised of an earlier 23andMe PD cohort [23], a recent 23andMe PD cohort [24], and a public PD GWAS from an additional 13 cohorts [25]. The genotyping and imputation procedures for each of these cohorts were described previously and strict quality control procedures were performed in each GWAS summary statistics data. We performed an inverse-variance weighted meta-analysis of these summary statistics totaling 38,426 patients with PD and 807,269 controls.

#### Selection of instrumental variables

We selected the top leading SNPs that were identified to be of genome-wide significance and independent of each other as instrumental variables for RHR (N = 76) and intelligence (N = 242). To reduce the possibility of mixing the effect alleles for ambiguous or palindromic SNPs, we used proxy SNPs that are not ambiguous or palindromic for the MR analysis (table e-1 and table e-2).

#### Mendelian randomization analysis

MR analysis was performed using the instrument variables described above. Effect sizes and standard errors were obtained for each SNP from the GWAS summary statistics for RHR and intelligence. The analysis was performed using the inverse variance weighted method and complemented with the MR-Egger regression [26] and weighted median [27] methods. The MR-Egger regression estimates the effects by adjusting for horizontal pleiotropy provided that other conditions of MR-Egger regression are satisfied. The weighted median regression calculates the effects that are robust as long as half or more of the SNPs are valid instruments. We examined the heterogeneity of the estimates by using a scatter plot and assessed the probable directional pleiotropy using a funnel plot. A leave-one-out sensitivity analysis was conducted by removing a single variant from the analysis in turn. The fluctuation of the estimates in response to excluding each variant reflects the possibility of outlier variant in the causal estimation. We also assessed the potential reverse causation in which PD may affect RHR and IQ using MR approaches. We used the *MendelianRandomization* and *TwoSampleMR* packages [28, 29] for the MR analysis.

We used Stata 15.1 (StataCorp, TX, USA) and R 3.6 (R Project for Statistical Computing) to perform the statistical analyses and used 2-tailed *P* value of < 0.05 as statistically significant.

#### Data availability statement

The data of the cohort study are not open access; however we welcome collaborations. Please contact Prof. Fang Fang (fang.fang@ki.se) for more information. The GWAS summary statistics for RHR and intelligence are publicly available. The GWAS summary statistics of PD are possible to obtain through 23andMe.

## Results

Table 1 shows the baseline characteristics of the cohort participants. During a mean follow-up of 11 years, a total of 1,034 conscripts were diagnosed with PD. The median age at diagnosis was 53 years (range: 40–63 years).

**Table 1:** Baseline characteristics of the cohort participants (N = 1,059,485)

Characteristics	N	%
Age at conscription examination (years)		
17	43,240	4.08
18	691,940	65.31
19	291,117	27.48
20	33,188	3.13
Calendar period of conscription examination		
1968–1980	509,547	48.09
1981–1993	549,938	51.91
Physical fitness (Watts)		
< 229	342,670	32.34
230–280	364,014	34.36
> 281	349,696	33.01
Missing	3,105	0.29
Body mass index (kg/m^2^)		
Underweight (< 18.5)	95,186	8.98
Normal (18.5–24.99)	848,596	80.10
Overweight (≥ 25)	103,242	9.74
Missing	12,461	1.18
Resting heart rate (beats per minute)		
Slow (< 60)	83,357	7.87
Normal (60–100)	557,683	52.64
Fast (> 100)	18,666	1.76
Missing	399,779	37.73
Blood pressure (mm Hg)		
Normal (< 120/80)	181,511	17.13
Elevated (120–129/ < 80)	302,185	28.52
Hypertension (≥ 130/80)	556,102	52.49
Missing	19,687	1.86
IQ (stanine)		
1–3	217,527	20.53
4–6	574,119	54.19
7–9	258,500	24.40
Missing	9,339	0.88
Stress resilience (stanine)		
1–3	186,323	17.59
4–6	622,427	58.75
7–9	220,985	20.86
Missing	29,750	2.80

Physical fitness was positively correlated with BMI (ρ = 0.30; *p < *0.001), blood pressure (ρ = 0.04; *p* < 0.001), IQ (ρ = 0.13; *p* < 0.001), and stress resilience (ρ = 0.31; *p < *0.001). Positive correlations were also noted between BMI and blood pressure (ρ = 0.15; *p < *0.001), BMI and stress resilience (ρ = 0.11; *p < *0.001), RHR and blood pressure (ρ = 0.24; *p < *0.001), blood pressure and IQ (ρ = 0.004; *p < *0.001), blood pressure and stress resilience (ρ = 0.02; *p < *0.001), and IQ and stress resilience (ρ = 0.38; *p < *0.001). Negative correlations were noted between physical fitness and RHR (ρ = -0.20; *p < *0.001), BMI and RHR (ρ = -0.03; *p < *0.001), BMI and IQ (ρ = -0.04; *p < *0.001), RHR and IQ (ρ = -0.05; *p < *0.001), as well as RHR and stress resilience (ρ = -0.17; *p < *0.001).

Table 2 presents the HRs of PD in relation to the studied attributes. Conscripts with a fast RHR had a higher risk of PD compared to conscripts with a normal RHR. Conscripts with an IQ above the highest tertile had also a higher risk of PD compared to conscripts with an IQ below the lowest tertile. No association was however noted for physical fitness, BMI, blood pressure, and stress resilience. The result pattern was similar between the simple model (adjusted for age, calendar period of conscription examination, and parental socioeconomic status) and the full model (additionally mutually adjusted for all attributes). The association of IQ with PD diminished slightly but remained statistically significant, after adjustment for adult education (Table 3). In contrast, adjusting for adult cardiovascular disease did not change the association of RHR with PD clearly. Among the 1034 conscripts diagnosed with PD, 996 of them had PD as main diagnosis during follow-up (96.34%). Sensitivity analyses using main diagnosis only as the definition for PD provided similar results as in the main analyses (table e-3 and table e-4). Starting the follow-up from 50 years of age (HR = 1.59; 95% CI = 1.10–2.29 for fast resting heart rate and HR = 1.62; 95% CI = 1.24–2.12 for IQ above the highest tertile) or including also early-onset PD in the definition of PD (HR = 1.53; 95% CI = 1.12–2.08 for fast resting heart rate and HR = 1.33; 95% CI = 1.08–1.63 for IQ above the highest tertile) yielded comparable results.

**Table 2:** Associations of physical and cognitive fitness in late adolescence with the future risk of Parkinson's disease (PD)

Characteristics	No. of PD	HR^a^ (95% CI)	*P*-value	HR^b^ (95% CI)	P-value
Physical fitness (Watts)					
< 232	597	1.00		1.00	
233–289	307	1.09 (0.94–1.26)	0.249	1.12 (0.96–1.30)	0.143
> 290	130	1.08 (0.87–1.32)	0.469	1.15 (0.91–1.46)	0.246
Body mass index (kg/m^2^)					
Underweight (< 18.5)	126	1.14 (0.95–1.38)	0.169	1.12 (0.91–1.36)	0.269
Normal (18.5–24.99)	799	1.00		1.00	
Overweight (≥ 25)	86	1.08 (0.87–1.35)	0.492	1.12 (0.89–1.41)	0.334
Resting heart rate (beats per minute)					
Slow (< 60)	111	1.03 (0.84–1.25)	0.771	0.99 (0.81–1.22)	0.923
Normal (60–100)	805	1.00		1.00	
Fast (> 100)	43	**1.44 (1.06–1.96)**	**0.020**	**1.52 (1.11–2.07)**	**0.008**
Blood pressure (mmHg)					
Normal (< 120/ < 80)	179	1.00		1.00	
Elevated (120–129/ < 80)	298	1.04 (0.87–1.26)	0.678	1.04 (0.86–1.26)	0.687
Hypertension (≥ 130/ ≥ 80)	534	0.94 (0.80–1.12)	0.471	0.91 (0.76–1.09)	0.305
IQ (stanine)					
1–3	172	1.00		1.00	
4–6	519	1.13 (0.95–1.35)	0.173	1.15 (0.96–1.38)	0.131
7–9	318	**1.40 (1.16–1.68)**	0.0003*	**1.46 (1.19–1.78)**	0.0002*
Stress resilience (stanine)					
1–3	209	1.00		1.00	
4–6	545	0.91 (0.77–1.06)	0.247	0.85 (0.72–1.00)	0.052
7–9	247	1.03 (0.85–1.23)	0.754	0.90 (0.74–1.11)	0.308

^a^HR: hazard ratio; adjusted for attained age, calendar period of conscription, and parental socioeconomic status

^b^HR: hazard ratio; adjusted for attained age, calendar period of conscription, parental socioeconomic status, physical fitness, body mass index, resting heart rate, blood pressure, IQ, and stress resilience

^*^P < *0.001*

**Table 3:** Associations of resting heart rate and IQ in late adolescence with the future risk of Parkinson's disease, after additional adjustment for adult education and cardiovascular diseases

Characteristics	HR (95% CI)	P-value
Resting heart rate (beats per minute)		
Slow (< 60)	0.98 (0.80–1.21)	0.848
Normal (60–100)	1.00	
Fast (> 100)	**1.52 (1.12–2.08)**	**0.008**
IQ (stanine)		
1–3	1.00	
4–6	1.12 (0.93–1.35)	0.233
7–9	**1.36 (1.09–1.68)**	0.005*

HR: hazard ratio; adjusted for attained age, calendar period of conscription, parental socioeconomic status, physical fitness, body mass index, resting heart rate, blood pressure, IQ, stress resilience, and adult education and cardiovascular diseases

^*^P < *0.01*

The results of MR analysis for RHR, intelligence, and PD risk are described in Table 4. No clear evidence was observed for an association of RHR with PD risk (odds ratio [OR]: 0.99, 95% CI: 0.98, 1.00, *P* = 0.17) using the inverse variance weighted method. The analyses from MR-Egger regression and weighted median analysis yielded similar results. Directional pleiotropy was not suggested for the SNPs of RHR according to the MR-Egger regression analysis (intercept: 0.001, 95% CI: -0.01, 0.01, *P* = 0.84). Higher intelligence was however associated with a higher risk of PD (OR: 1.19, 95% CI: 1.06, 1.35, *P* = 0.004) using the inverse variance weighted method. The point estimates from MR-Egger regression and weighted median analysis were in the same direction, although with wider CIs. No strong evidence was observed for directional pleiotropy for the SNPs of intelligence from the MR-Egger regression analysis (intercept: − 0.006, 95% CI: − 0.017, 0.004, *P* = 0.23). We did not find significant reverse associations of PD with RHR or IQ (table e-5). The scatter plot, leave-one-out analysis, and funnel plot were presented in the figures e-1–12 for each MR analysis.

**Table 4:** Associations of resting heart rate and intelligence with the risk of Parkinson’s disease, a Mendelian randomization analysis

	OR (95% CI)	*P* value
Resting heart rate		
IVW	0.99 (0.98, 1.00)	0.174
Weighted median	0.99 (0.98, 1.00)	0.240
MR-Egger	0.99 (0.96, 1.02)	0.478
MR-Egger intercept	0.001 (-0.010, 0.012)	0.837
Intelligence		
IVW	1.19 (1.06, 1.35)	0.004
Weighted median	1.22 (1.07, 1.40)	0.004
MR-Egger	1.63 (0.96, 2.75)	0.068
MR-Egger intercept	− 0.006 (− 0.017, 0.004)	0.231

OR: odds ratio; IVW: inverse variance weighted; MR: Mendelian randomization

## Discussion

In a cohort study including more than one million Swedish men participating in conscription examination, we found statistically significant associations of higher RHR and higher IQ measured at late adolescence with a higher risk of PD later in life, after adjusting for multiple potential confounders. We found however no association of physical fitness, BMI, blood pressure, and stress resilience with the risk of PD. The association of RHR with PD was not attributable to adult cardiovascular disease. The association of IQ with PD diminished slightly but remained statistically significant after additional adjustment for adult education. The MR analysis used GWAS summary statistics from more than 800,000 individuals and provided additional evidence to support a potentially causal relationship between IQ and PD, but not between RHR and PD.

### Comparisons with other studies

Our finding that higher RHR was associated with a higher risk of PD is consistent with previous findings that decreased cardiovascular response—maximum heart rate—after cardiac stress test [9], ECG abnormalities and carotid stenosis [8], and lower heart rate variability [10] were all associated with a higher risk of PD. In the large prospective Atherosclerosis Risk in Communities (ARIC) study including 12,162 participants, decreased heart rate variability was found to be associated with a higher risk of PD during a 20 years’ follow-up period [10]. Similar result was however not observed in the other two smaller exploratory studies, which could partially be due to limited statistical power [8, 9]. Although RHR, maximum heart rate, electrocardiogram (ECG) abnormalities, and heart rate variability describe different capacities of the heart and are used for different purposes in practice, they are all inter-related and represent collectively the autonomic nervous functions of the heart. RHR and heart rate variability demonstrate for instance, mathematically, an inverse relationship and have a strong genetic correlation (*r* = − 0.55 ~ − 0.77) [30]. Because cardiac function impairment has been suggested as the earliest stage of PD [7] and because a causal relationship between RHR and PD was not supported by the MR analysis, the noted association of RHR with PD might suggest instead that altered cardiac autonomic function is already existent before 20 years of age in PD.

Our finding that higher IQ was associated with a higher risk of PD extends the results of previous studies that showed a higher risk of PD among people with higher educational attainment or socioeconomic status [31–33]. While IQ is highly correlated with educational attainment and socioeconomic status [22, 34–36], the association of IQ with PD per se has not been studied. In the present study, we found the association of IQ with PD to be robust and independent of multiple potential confounders including age, parental socioeconomic status, physical fitness, BMI, RHR, blood pressure, and stress resilience. Additional adjustment for adult educational attainment only slightly diminished the association, suggesting that the association of IQ at late adolescence with PD is unlikely completely attributable to adult educational achievement. The MR analysis yielded similar result, further suggesting a potentially causal relationship between IQ and PD and corroborating previous studies examining a link between cognitive performance and PD [25]. Although very little is known today regarding the underlying mechanisms, a link between IQ and PD is plausible [32]. For example, individuals with higher IQ or educational levels have been shown to have lower levels of cholesterol [37], which have been repeatedly associated with increased risk of PD [38–44], and lower likelihood of smoking [45, 46], which is a known protective factor for PD [1]. Regardless, because IQ is a complex modality to quantify and may be affected by additional factors not accounted for or discussed in the present study, future studies are needed to validate this finding. If confirmed, additional efforts are needed to examine potential underlying mechanisms linking together IQ and PD.

In this study, we did not find clear association of physical fitness with the risk of PD. A previous study used metabolic equivalents (METs) estimated from peak treadmill speed and grade as a measurement for physical fitness among 7,347 veterans and found that veterans with a higher physical fitness (METs > 12) had a lower risk of PD, compared to veterans with lower physical fitness (METs < 8) (HR: 0.22, 95% CI: 0.10–0.49) [3]. The association was however non-existent when treating METs as a continuous variable or after additionally adjusting for age and smoking (HR: 1.07; 95% CI: 0.98–1.16). Another study, using a small proportion of the men included in the Swedish Coscript Register, found an association between lower muscle strength at 18 years of age and a higher risk of PD, but also failed to demonstrate an association between physical fitness and PD.^47^ Because physical fitness is positively correlated with muscle strength^47^ whereas negatively correlated with RHR as shown in the present study, a null association between physical fitness and PD is surprising. Future studies using other instruments in measuring physical fitness are therefore needed to further examine this relationship. Our finding that BMI in late adolescence was not associated with the risk of PD later on corroborates findings from a recent meta-analysis of previous prospective cohort studies, which found little evidence to support BMI as a risk factor for PD [5]. This is however in conflict with a recent MR analysis, which suggested an inverse association between BMI and PD [6]. The precise reasons for these conflicting findings remain unknown.

The present study did not provide strong evidence to link blood pressure in late adolescence and PD. A recent meta-analysis found a positive association of hypertension with PD in cohort studies, whereas an inverse association between hypertension and PD in case–control studies [11]. The authors speculated that the inverse association noted in case–control studies might be an artefact and could be due to the fact that PD patients with hypertension were less likely to participate in a study, compared to PD-free individuals (i.e. controls) with hypertension, leading to a lower-than-expected prevalence of hypertension in patients with PD [11]. If the positive association suggested by the cohort studies turns out true, it might also represent a sign of cardiac alteration in premotor PD, rather than a cause of PD, as does RHR. The lack of association between blood pressure and PD in the present study might instead indicate that PD-related blood pressure alteration has a later debut compared to RHR. In line with these findings, other symptoms of autonomic dysfunction concerning heart, bladder, intestines, etc. might also need to be studied in the pre-motor stage of PD, in addition to RHR and blood pressure. We found no association between stress resilience and the subsequent risk of PD, and this is the first study to our best knowledge to investigate the role of stress resilience in PD.

### Strengths and weaknesses of the study

The present study is the largest to date to investigate the associations of important correlates of physical activity, measured in late adolescence, with the future risk of PD. The prospective study design, the large sample size, the long and complete follow-up period, and the large number of PD cases all ensured a robust analysis with high statistical power. The independent collections of the exposures and the outcome greatly alleviated concerns about information and selection biases. The additional adjustment of a rich panel of potential confounders and mediators (adult education and cardiovascular disease) constitutes another strength. Finally, the combined use of observational study and MR analysis is a novelty, which provides additional evidence for a potentially causal relationship between IQ and PD.

Our study has several limitations. First, because the study cohort is based on a Scandinavian population of men and the GWAS summary statistics are from the European ancestry, our results may not be directly generalizable to women or other ethnic populations. Second, we did not have data on other risk factors for PD, such as smoking and cholesterol levels [1]. Although the relationship between IQ and PD is not likely greatly confounded by these factors because MR analysis is less prone to residual confounding as observational studies, the potential roles of these factors in mediating the link between IQ and PD need to be studied further. The maximum age at follow-up was 65 years in the present study and as a result we focused on a group of patients with PD diagnosed at relatively early age (mean age at diagnosis = 53). Because majority of the PD patients are diagnosed above 60 years of age, the present findings are not directly generalizable to PD in general. Because patients with PD were ascertained through inpatient care records alone before 2001, we were likely to have missed patients that were never hospitalized—for PD specifically or for any other reasons—before 2001. This was also reflected by the relatively low sensitivity of PD definition based on inpatient care records alone in the Swedish Patient Register [20]. Such ascertainment problem is however believably much more limited after 2001 when we identified patients with PD through both inpatient and outpatient care records. Further, such misclassification of outcome is likely non-differential in terms of exposure (e.g., resting heart rate or IQ at late adolescence), and will mostly lead to an under-estimated association between the exposure and outcome [47], 48]. Further, although the positive predictive value of PD definition based on the inpatient care records of the Swedish Patient Register is satisfactory, the corresponding value based on outpatient specialist care records remains unknown. Some of the PD cases identified during follow-up might have therefore been misclassified as PD patients. Further, we did not correct for multiple testing in the cohort analysis, because we sought to replicate our findings using MR approach in an independent sample. Finally, the MR analysis was based on GWAS summary statistics rather than individual-level genotype data. On the one hand, this analysis strategy maximizes the utility of published GWAS data, whereas on the other hand, the potential gene-environment interactions, non-linear relationships, and potential violations of MR assumptions cannot be tested. The latter two concerns are however not greatly probable because our cohort analysis did not reveal strong evidence for a non-linear association and MR-Egger regression analysis (*intercepts*) did not suggest notable pleiotropic effects of the selected SNPs.

## Conclusions

Taken together, our study, combing both individual data from a large prospective cohort and a Mendelian randomization analysis using GWAS summary statistics, provides novel evidence for a potentially causal relationship between IQ and PD. The association of RHR in late adolescence with PD risk might instead suggest that cardiac autonomic function impairment might have started before 20 years of age in PD.

## Supplementary Information

Below is the link to the electronic supplementary material.

Supplementary file1 (DOCX 73 KB)

Supplementary file2 (PDF 8 KB)

Supplementary file3 (PDF 15 KB)

Supplementary file4 (PDF 9 KB)

Supplementary file5 (PDF 9 KB)

Supplementary file6 (PDF 8 KB)

Supplementary file7 (PDF 17 KB)

Supplementary file8 (PDF 9 KB)

Supplementary file9 (PDF 9 KB)

Supplementary file10 (PDF 6 KB)

Supplementary file11 (PDF 7 KB)

Supplementary file12 (PDF 6 KB)

Supplementary file13 (PDF 6 KB)

## Data Availability

The GWAS summary statistics can be obtained from the 23andMe research team under a data transfer agreement.

## Code availability

The codes were written in SAS and R and will be available upon request.
