# Supplementary material for: Heart rate, intelligence in adolescence, and Parkinson’s disease later in life
Source: Eur J Epidemiol. 2021 Mar 6;36(10):1055–64. doi: 10.1007/s10654-021-00730-y (PMC8542538; doi:10.1007/s10654-021-00730-y)
Supplement: Supplementary file 1 — Supplementary file1 (DOCX 73 KB) [file 10654_2021_730_MOESM1_ESM.docx]

**Supplementary Tables**

**Table e-1.** SNPs selected for resting heart rate

| rsID | chr | pos | A1 | A2 | beta | se |
| --- | --- | --- | --- | --- | --- | --- |
| rs12073932 | 1 | 6293890 | G | A | 0.214049 | 0.105324 |
| rs186315294 | 1 | 9934641 | G | A | 0.920078 | 0.196911 |
| rs272564 | 1 | 45012273 | A | C | 0.40682 | 0.0485046 |
| rs2152735 | 1 | 87893132 | G | A | -0.316226 | 0.0460348 |
| rs41317993 | 1 | 207961732 | G | A | 0.598281 | 0.0701549 |
| rs12408022 | 1 | 217718789 | C | T | 0.280413 | 0.0493125 |
| rs1260326 | 2 | 27730940 | T | C | -0.286787 | 0.0435462 |
| rs12713404 | 2 | 60006705 | G | T | -0.257367 | 0.0456674 |
| rs62144050 | 2 | 66725062 | T | C | 0.265893 | 0.0474268 |
| rs17494056 | 2 | 160052731 | A | C | -0.270045 | 0.0483712 |
| rs1376875 | 2 | 175568719 | C | T | -0.281308 | 0.0580556 |
| rs151041685 | 2 | 179725237 | G | T | 1.10808 | 0.0760156 |
| rs62172372 | 2 | 188242369 | A | G | 0.373803 | 0.0547583 |
| rs907683 | 2 | 220299541 | G | T | -0.353527 | 0.0442015 |
| rs4608502 | 2 | 228134155 | T | C | 0.268144 | 0.0459663 |
| rs13002735 | 2 | 232268884 | A | C | -0.36731 | 0.050332 |
| rs73070981 | 3 | 38631838 | T | C | -0.376519 | 0.0602223 |
| rs3749237 | 3 | 49770032 | G | A | 0.330021 | 0.0457024 |
| rs2358740 | 3 | 53455569 | G | T | -0.263127 | 0.045805 |
| rs1483890 | 3 | 69410725 | A | G | 0.291817 | 0.0465226 |
| rs11920570 | 3 | 122090102 | G | A | 0.365583 | 0.0483261 |
| rs7612445 | 3 | 179172979 | G | T | -0.389209 | 0.0545484 |
| rs4422407 | 4 | 23978109 | T | C | 0.193825 | 0.0430445 |
| rs1425518 | 4 | 143462437 | C | T | -0.316683 | 0.0533395 |
| rs6845865 | 4 | 148974602 | T | C | -0.381374 | 0.0574402 |
| rs1921102 | 5 | 30896358 | A | C | -0.257263 | 0.0434817 |
| rs1468333 | 5 | 137552970 | T | C | -0.270072 | 0.0444438 |
| rs4868243 | 5 | 172643118 | G | A | -0.381279 | 0.0580271 |
| rs2744373 | 6 | 7554527 | G | A | 0.32895 | 0.0595594 |
| rs236349 | 6 | 36820565 | A | G | 0.286882 | 0.0453208 |
| rs9401060 | 6 | 118561348 | A | G | 0.515198 | 0.0429713 |
| rs1320761 | 6 | 122168138 | C | T | 0.891253 | 0.068278 |
| rs58437978 | 7 | 35258277 | T | C | -0.273544 | 0.0431229 |
| rs180242 | 7 | 93549596 | T | A | -0.254391 | 0.0448455 |
| rs17881696 | 7 | 100493359 | G | A | 0.588916 | 0.0556519 |
| rs41748 | 7 | 116446573 | T | G | -0.242047 | 0.0430606 |
| rs3944151 | 7 | 126970005 | A | G | -0.306055 | 0.0480928 |
| rs34990159 | 7 | 130964537 | G | A | -0.293083 | 0.0431243 |
| rs73158705 | 7 | 136576100 | A | G | 0.403759 | 0.0580575 |
| rs56233017 | 8 | 144981488 | G | A | -0.683836 | 0.10531 |
| rs748802 | 9 | 35909518 | G | A | -0.275715 | 0.045686 |
| rs1885427 | 9 | 99188248 | A | G | -0.286583 | 0.0525333 |
| rs10739663 | 9 | 128278739 | A | G | -0.290655 | 0.0428612 |
| rs12576326 | 11 | 44980383 | A | G | 0.273363 | 0.0451369 |
| rs174536 | 11 | 61551927 | A | C | 0.396942 | 0.0448825 |
| rs75190942 | 11 | 128764571 | C | A | -0.495038 | 0.0752319 |
| rs11062107 | 12 | 2187041 | A | G | -0.420948 | 0.057668 |
| rs10841486 | 12 | 20472202 | T | C | -0.299773 | 0.0520826 |
| rs4963772 | 12 | 24758480 | G | A | -0.733107 | 0.0596177 |
| rs1050288 | 12 | 27955296 | C | T | -0.257599 | 0.0456672 |
| rs1994135 | 12 | 33682405 | T | C | 0.404996 | 0.0426145 |
| rs10880689 | 12 | 37930102 | A | G | 0.199932 | 0.0436527 |
| rs867400 | 12 | 64976850 | T | C | 0.296168 | 0.0432819 |
| rs12579753 | 12 | 82219376 | C | T | -0.281746 | 0.0512754 |
| rs12889267 | 14 | 21542766 | A | G | 0.413297 | 0.0576767 |
| rs422068 | 14 | 23864804 | T | C | 0.779885 | 0.0443492 |
| rs17780385 | 14 | 72889376 | C | T | -0.381533 | 0.0719576 |
| rs1549118 | 14 | 78379684 | C | T | 0.261376 | 0.04781 |
| rs17201923 | 14 | 85796564 | A | G | -0.46605 | 0.0477788 |
| rs4900069 | 14 | 91583373 | A | C | 0.249781 | 0.0441629 |
| rs7174098 | 15 | 73664041 | G | A | -0.610093 | 0.0586888 |
| rs1592560 | 16 | 10811687 | A | C | -0.248269 | 0.0451161 |
| rs3915499 | 16 | 15910743 | G | A | 0.316194 | 0.0459467 |
| rs7194801 | 16 | 65286870 | T | C | -0.325788 | 0.0434793 |
| rs79121763 | 17 | 15195279 | C | T | -0.516832 | 0.0766183 |
| rs12941356 | 17 | 17716531 | A | G | -0.251388 | 0.0431355 |
| rs117159291 | 18 | 20129524 | A | C | 0.564208 | 0.100417 |
| rs11083258 | 18 | 25766218 | A | C | -0.329834 | 0.0570112 |
| rs11081761 | 18 | 30003322 | G | A | -0.396288 | 0.0608081 |
| rs61735998 | 18 | 34289285 | G | T | -0.982606 | 0.138673 |
| rs16974196 | 19 | 40833470 | G | A | 0.260984 | 0.0459554 |
| rs56131196 | 19 | 45422846 | G | A | -0.306061 | 0.0549505 |
| rs6123471 | 20 | 36840156 | T | C | -0.651568 | 0.0427694 |
| rs17265513 | 20 | 39832628 | T | C | 0.30313 | 0.0542885 |
| rs2283847 | 22 | 28181399 | C | T | -0.244623 | 0.0445704 |
| rs2076028 | 22 | 39150450 | G | A | -0.362174 | 0.0472559 |

**Table e-2.** SNPs selected for intelligence

| **chr** | **pos** | **A1** | **A2** | **beta** | **se** | **rsID** |
| --- | --- | --- | --- | --- | --- | --- |
| 1 | 22425642 | T | C | 0.0242133375220269 | 0.00404905309732891 | rs10917152 |
| 1 | 32106494 | A | G | -0.0199794775401961 | 0.00281758250461092 | rs7546297 |
| 1 | 41750648 | A | C | -0.0269917353038689 | 0.00331227577664362 | rs12035012 |
| 1 | 41835633 | A | G | 0.0176572588137298 | 0.00281435428972423 | rs4636447 |
| 1 | 44014280 | T | C | 0.016411388119762 | 0.00285813098567781 | rs2842188 |
| 1 | 44250301 | T | G | -0.0288431308792363 | 0.00444561203440757 | rs4660749 |
| 1 | 59560337 | T | C | -0.0172034627415813 | 0.00276227725458916 | rs1831539 |
| 1 | 69284323 | T | C | 0.0258700018485373 | 0.00446342336931285 | rs112134147 |
| 1 | 72588119 | A | G | -0.0275414793525812 | 0.00317590859693048 | rs12128707 |
| 1 | 72749848 | T | C | -0.0317270048438643 | 0.00341738526969671 | rs3128341 |
| 1 | 84417585 | T | C | -0.10782328442011 | 0.0169853945211264 | rs41293013 |
| 1 | 96199657 | T | C | -0.0214579674561173 | 0.00273419564935235 | rs6668048 |
| 1 | 98498441 | A | G | 0.0340493233670521 | 0.00548121754137992 | rs11804556 |
| 1 | 98588885 | T | C | 0.0155576244725347 | 0.00273757249208776 | rs1473474 |
| 1 | 1,04E+08 | T | C | -0.0181941258887876 | 0.00277434063567971 | rs1528204 |
| 1 | 1,1E+08 | A | G | -0.0191116442707662 | 0.00298712789477434 | rs1144593 |
| 1 | 1,54E+08 | A | G | -0.0182837525085779 | 0.00309946643644311 | rs112780312 |
| 1 | 1,72E+08 | T | C | 0.0177069435308973 | 0.00352376985689498 | rs2223602 |
| 1 | 1,82E+08 | T | C | 0.0200505559184013 | 0.00362381274505716 | rs199928 |
| 1 | 2,02E+08 | T | C | 0.0187864011993969 | 0.00304627877402253 | rs2678210 |
| 1 | 2,17E+08 | A | G | 0.0163751200356413 | 0.00292517328253685 | rs10779271 |
| 2 | 23934816 | T | C | -0.0171675156564887 | 0.0030208544178231 | rs12470949 |
| 2 | 41616346 | T | C | -0.0179756831886435 | 0.00292715896248876 | rs967569 |
| 2 | 44116836 | T | C | -0.0149146942322597 | 0.00273413276485055 | rs2955280 |
| 2 | 45171546 | T | C | -0.0182467024690902 | 0.00334249907841916 | rs62131236 |
| 2 | 51250505 | T | C | 0.0159272436336934 | 0.0029016658104743 | rs7557525 |
| 2 | 60499463 | A | G | -0.0276825953956288 | 0.00465175523367985 | rs58593843 |
| 2 | 60713235 | A | G | 0.0189954509400107 | 0.00274977575854237 | rs10189857 |
| 2 | 64306984 | A | G | -0.0194098346279653 | 0.0032055878824055 | rs2576835 |
| 2 | 71539301 | T | C | -0.0207869352217104 | 0.00274741411865059 | rs4852252 |
| 2 | 73565293 | A | G | -0.0179806869445597 | 0.00300780979333552 | rs11898362 |
| 2 | 82444107 | T | C | 0.0160859385462951 | 0.00274504070755889 | rs11678106 |
| 2 | 1,01E+08 | T | C | 0.0228351560060748 | 0.00284479332329323 | rs2309812 |
| 2 | 1,01E+08 | A | G | 0.0480963232623181 | 0.0076598699255165 | rs71413877 |
| 2 | 1,18E+08 | T | C | 0.0159491751618673 | 0.00282536318190741 | rs60262711 |
| 2 | 1,38E+08 | T | G | -0.015631792598654 | 0.00277356149727716 | rs2558096 |
| 2 | 1,44E+08 | A | G | -0.0193366001089599 | 0.00285284746370019 | rs10189912 |
| 2 | 1,56E+08 | A | G | -0.0165882458236817 | 0.00277999762421346 | rs3106666 |
| 2 | 1,57E+08 | A | G | 0.0260267144450988 | 0.00442706488264991 | rs72906053 |
| 2 | 1,57E+08 | A | C | 0.0190504200101145 | 0.0027458085918297 | rs6436555 |
| 2 | 1,61E+08 | A | G | -0.0160525855471536 | 0.00274403171746215 | rs10192369 |
| 2 | 1,62E+08 | T | C | 0.0277466532394114 | 0.00486612648884801 | rs17221725 |
| 2 | 1,62E+08 | T | C | 0.020221206644403 | 0.00276548230913608 | rs3754970 |
| 2 | 1,63E+08 | T | C | 0.0207847924893078 | 0.00274858403720019 | rs2268894 |
| 2 | 1,64E+08 | A | C | 0.0179018564105143 | 0.00305179959265502 | rs3956504 |
| 2 | 1,67E+08 | T | G | 0.0171704403553948 | 0.0029028639654091 | rs62174851 |
| 2 | 1,72E+08 | A | G | -0.0192572909165477 | 0.00332194081706877 | rs3749034 |
| 2 | 1,81E+08 | A | C | -0.0207086206313565 | 0.0037597350456348 | rs10196283 |
| 2 | 1,81E+08 | T | C | 0.0211458118892426 | 0.00351142675012332 | rs62181012 |
| 2 | 1,86E+08 | A | G | 0.0190651433149765 | 0.00322536682709804 | rs62198803 |
| 2 | 1,99E+08 | A | C | -0.0155782349095149 | 0.00284430069554774 | rs11679484 |
| 2 | 2E+08 | A | G | -0.0161756831719628 | 0.00276933456119891 | rs1455344 |
| 2 | 2E+08 | T | C | 0.0218376068193685 | 0.00365789058950897 | rs35731967 |
| 2 | 2,32E+08 | A | G | -0.0166606670394936 | 0.00287898168990731 | rs13024268 |
| 3 | 16858468 | T | G | -0.0248376422578917 | 0.00405711242370005 | rs73139272 |
| 3 | 24062661 | A | G | -0.0248092053718445 | 0.0029287221546269 | rs6550835 |
| 3 | 35538452 | A | G | 0.0170937545202073 | 0.00275928240842733 | rs1589652 |
| 3 | 48675856 | A | C | 0.0236135643558805 | 0.00431771152969107 | rs13096357 |
| 3 | 48708347 | T | C | 0.026184323518469 | 0.00435389483180395 | rs73078367 |
| 3 | 49890613 | T | C | -0.0308409353279913 | 0.00275144395824706 | rs2352974 |
| 3 | 50463935 | T | C | 0.0276465486944033 | 0.00463557154500391 | rs1540293 |
| 3 | 52563718 | T | C | 0.0193434611877398 | 0.00274765073689485 | rs4687625 |
| 3 | 54234722 | A | G | 0.0188778729394742 | 0.0033400341364958 | rs4485754 |
| 3 | 71545170 | A | C | 0.0183255062648826 | 0.00277323036696165 | rs11720523 |
| 3 | 85171415 | A | G | -0.0449612601692593 | 0.00686431452965791 | rs6770622 |
| 3 | 89587262 | A | G | 0.0165331147131428 | 0.00279937600967538 | rs7652296 |
| 3 | 93537923 | A | G | -0.0173724151112814 | 0.00305637141296294 | rs62266110 |
| 3 | 1,08E+08 | T | C | 0.0188537448207771 | 0.00340197488646284 | rs3860537 |
| 3 | 1,36E+08 | A | G | -0.0175188839724647 | 0.00291932744083731 | rs9853960 |
| 3 | 1,37E+08 | T | C | 0.0180950826936895 | 0.00291715020049807 | rs13071190 |
| 3 | 1,4E+08 | A | G | 0.0226956171762162 | 0.00368495164413318 | rs59142272 |
| 3 | 1,42E+08 | A | G | 0.0205064102926081 | 0.00384447137094265 | rs9942 |
| 4 | 696848 | T | C | 0.0251275007963291 | 0.00421531635570023 | rs12646225 |
| 4 | 2717690 | T | C | -0.0163990355623148 | 0.00275336392919994 | rs2295499 |
| 4 | 16286266 | T | C | 0.017362920566882 | 0.00314374806570379 | rs60333108 |
| 4 | 17259908 | A | G | 0.0154910586568945 | 0.00283979077119972 | rs144246 |
| 4 | 17823902 | T | C | 0.0274835702989251 | 0.00381028286412382 | rs11932971 |
| 4 | 25408838 | A | G | 0.0289955369300314 | 0.0035938940171085 | rs34811474 |
| 4 | 65785318 | T | C | -0.016590103556161 | 0.00321264592489562 | rs62312751 |
| 4 | 67970101 | A | G | -0.0197960827467591 | 0.00272861237033207 | rs6819372 |
| 4 | 94579640 | A | G | -0.0175560946028069 | 0.0029304113842108 | rs1972860 |
| 4 | 98092307 | A | C | 0.0185520173767131 | 0.00328994810723765 | rs4459994 |
| 4 | 1,03E+08 | T | C | -0.0526272894000899 | 0.0055432156520002 | rs13107325 |
| 4 | 1,03E+08 | A | G | -0.0162424353094865 | 0.00293821188666544 | rs62327949 |
| 4 | 1,06E+08 | A | G | -0.028280110622552 | 0.0028565768305608 | rs2726491 |
| 4 | 1,48E+08 | A | G | -0.0165875636147855 | 0.00296630250622058 | rs6840804 |
| 4 | 1,53E+08 | A | G | 0.019647497546139 | 0.0027341354781713 | rs6535809 |
| 5 | 7519298 | A | G | 0.0183752326640686 | 0.00325686505921103 | rs17826816 |
| 5 | 13100175 | A | G | 0.0163418933349756 | 0.00288267654524177 | rs1840847 |
| 5 | 26880925 | A | G | 0.0256356177930121 | 0.0044653575671507 | rs75973558 |
| 5 | 57657757 | T | G | 0.0180237378312598 | 0.0032133602836976 | rs13157057 |
| 5 | 59597487 | T | C | -0.0198190178449314 | 0.00353974242631388 | rs34426618 |
| 5 | 60960727 | T | C | 0.01596853525467 | 0.00278780294250524 | rs36033 |
| 5 | 62991802 | T | G | -0.0173389334491049 | 0.00276670391720199 | rs1812587 |
| 5 | 64020316 | T | G | 0.0453806088956589 | 0.00737776116008111 | rs80170948 |
| 5 | 88015545 | A | C | 0.0210490490361087 | 0.00276742690456334 | rs34316 |
| 5 | 89353210 | A | G | 0.024334073323318 | 0.00359918256520011 | rs166820 |
| 5 | 92562015 | A | G | 0.0164698008566883 | 0.00279813130422839 | rs11742251 |
| 5 | 1,03E+08 | A | G | -0.0417115589428552 | 0.00725166184681071 | rs76160968 |
| 5 | 1,08E+08 | A | C | 0.0153064541018193 | 0.00274358381462974 | rs12187824 |
| 5 | 1,09E+08 | T | C | 0.0152089066411581 | 0.00288813267017815 | rs7726343 |
| 5 | 1,11E+08 | T | C | 0.0205566415836436 | 0.00277155744689815 | rs1145123 |
| 5 | 1,14E+08 | T | G | -0.0158675006508439 | 0.00299669511819527 | rs283778 |
| 5 | 1,4E+08 | A | G | 0.0190654828691672 | 0.00273222741031344 | rs4463213 |
| 5 | 1,4E+08 | A | G | -0.0174954294469142 | 0.00275301800895581 | rs778591 |
| 5 | 1,65E+08 | A | G | 0.0155397323779828 | 0.00281925478555565 | rs830383 |
| 5 | 1,69E+08 | T | C | 0.0202624643090985 | 0.00347614759119892 | rs6860963 |
| 5 | 1,71E+08 | A | C | -0.0184232897765388 | 0.00321131075066041 | rs13184816 |
| 5 | 1,77E+08 | A | G | -0.0188286457596755 | 0.00279896621966337 | rs2450333 |
| 6 | 3451048 | T | C | -0.0171108145699373 | 0.00278496330890907 | rs9503599 |
| 6 | 11543342 | A | G | -0.0187160924051075 | 0.00293539717771448 | rs566237 |
| 6 | 13775907 | T | C | -0.0161490877801375 | 0.00293299814386805 | rs6459098 |
| 6 | 20893247 | A | C | 0.0163420062669458 | 0.00278114470165858 | rs6456379 |
| 6 | 21956404 | A | G | 0.0177580465248669 | 0.00297504548917186 | rs6903716 |
| 6 | 26529890 | A | G | 0.027210930166715 | 0.00498095005797456 | rs35433030 |
| 6 | 28712247 | A | G | -0.023797544301211 | 0.00390251628422614 | rs1233578 |
| 6 | 76536333 | A | C | 0.0149926126825972 | 0.00272890656763692 | rs1280049 |
| 6 | 98204498 | T | C | 0.0282436411981118 | 0.004936836426868 | rs77418166 |
| 6 | 98337903 | A | G | 0.0202031940536334 | 0.00295066365614626 | rs6928545 |
| 6 | 98550289 | A | C | 0.0316615554104482 | 0.0027414975677936 | rs1906252 |
| 6 | 99284532 | T | C | -0.0189850836324976 | 0.00292934479748458 | rs3823036 |
| 6 | 1,09E+08 | T | C | -0.0267241482624646 | 0.00278347549864228 | rs9384679 |
| 6 | 1,27E+08 | T | G | -0.0183679322158425 | 0.00324178118881795 | rs13212044 |
| 6 | 1,57E+08 | A | G | -0.0188666454127011 | 0.00307474664483395 | rs287879 |
| 7 | 8109522 | A | G | -0.0165316740919838 | 0.00273612613240381 | rs4725065 |
| 7 | 24177191 | T | C | 0.0160961673902343 | 0.00281401527801298 | rs115064 |
| 7 | 32484844 | T | C | 0.0160057306368754 | 0.00293629254024498 | rs59938271 |
| 7 | 44769190 | T | C | 0.0184149849243339 | 0.00275921260478482 | rs799444 |
| 7 | 69765505 | A | G | 0.0253803455775723 | 0.00417645969681953 | rs12698891 |
| 7 | 69948241 | A | G | 0.0176445940754208 | 0.00278393721606513 | rs13223152 |
| 7 | 69976237 | A | G | -0.0303381543819053 | 0.00522441094918294 | rs11972637 |
| 7 | 71759069 | A | C | -0.021968230323057 | 0.00274705893748369 | rs56150095 |
| 7 | 1,05E+08 | A | G | 0.017620892722817 | 0.00294516007401253 | rs7809847 |
| 7 | 1,27E+08 | A | G | -0.0192142825321975 | 0.0027915563754464 | rs4731365 |
| 7 | 1,27E+08 | A | G | 0.0188101887346829 | 0.00336136324779894 | rs4731368 |
| 7 | 1,28E+08 | A | G | -0.0217411997750718 | 0.00297457925503788 | rs4731392 |
| 7 | 1,28E+08 | A | G | 0.018956738554546 | 0.00312250676240257 | rs1043595 |
| 7 | 1,33E+08 | A | C | 0.0209452315395795 | 0.00273365068383966 | rs1362739 |
| 8 | 14002020 | T | G | -0.0201328534804042 | 0.00274813724821242 | rs13253386 |
| 8 | 20915316 | A | G | -0.0180868564953548 | 0.00297824081925816 | rs1473634 |
| 8 | 31019597 | T | C | -0.0163773876632988 | 0.00276178544069119 | rs10954779 |
| 8 | 66440593 | T | G | 0.0150707366030784 | 0.00275465849078385 | rs13276212 |
| 8 | 93180965 | T | C | -0.0247422345553296 | 0.00325170647329867 | rs2920940 |
| 8 | 1,05E+08 | A | G | 0.015490912651674 | 0.00275296119631669 | rs2111490 |
| 8 | 1,43E+08 | A | G | -0.0177529219558276 | 0.00289890952903783 | rs1106761 |
| 8 | 1,43E+08 | T | C | -0.015672682278109 | 0.00299898244893015 | rs4917225 |
| 8 | 1,43E+08 | A | G | 0.0173172152440614 | 0.00277742024764416 | rs4976976 |
| 8 | 1,46E+08 | T | C | -0.0162330969651138 | 0.00273376506653988 | rs2721173 |
| 9 | 23344737 | T | C | -0.0187977957442701 | 0.00305307710642685 | rs10757416 |
| 9 | 23362311 | T | G | 0.027833789393045 | 0.00280441202952595 | rs11793831 |
| 9 | 23805569 | T | C | -0.0198308385367846 | 0.00287195344486381 | rs702222 |
| 9 | 98216876 | A | G | -0.0163542694171977 | 0.00288944689349783 | rs28620532 |
| 9 | 99252464 | A | G | -0.0195312002132942 | 0.00348149736422357 | rs1057687 |
| 9 | 1,32E+08 | T | C | 0.0197245431697079 | 0.00302570074700229 | rs913264 |
| 9 | 1,35E+08 | T | C | 0.0157085810401722 | 0.00328425277862685 | rs2987389 |
| 10 | 29569272 | A | C | 0.022531564265753 | 0.00389752019819286 | rs7069887 |
| 10 | 65133156 | A | C | -0.0187089462299694 | 0.00296262014726356 | rs2393967 |
| 10 | 93442379 | T | C | 0.0154143735649154 | 0.00278640158440264 | rs1891273 |
| 10 | 1,02E+08 | T | C | 0.0160494342193201 | 0.00274819079097948 | rs1408579 |
| 10 | 1,04E+08 | A | G | 0.0201037098558291 | 0.00272962795055384 | rs749694 |
| 10 | 1,04E+08 | T | G | -0.0179806705225173 | 0.00281475743934209 | rs12772375 |
| 10 | 1,06E+08 | A | G | -0.0153148123817982 | 0.00277191174331189 | rs3896224 |
| 10 | 1,25E+08 | A | G | -0.0180893665151504 | 0.00293706226906159 | rs35608616 |
| 10 | 1,34E+08 | A | G | 0.0181920999942232 | 0.00322955796098405 | rs7921305 |
| 11 | 47606483 | A | G | -0.0166074362462872 | 0.00289580405340667 | rs11605348 |
| 11 | 63861317 | A | G | 0.01551210816401 | 0.00284104545128387 | rs7941785 |
| 11 | 79162622 | A | G | -0.0163231649872714 | 0.0028865013240091 | rs2373353 |
| 11 | 95594301 | A | G | 0.015741379722143 | 0.00288198090848462 | rs1271744 |
| 11 | 1,06E+08 | T | C | 0.0157066000915244 | 0.00284230910089113 | rs7116046 |
| 11 | 1,13E+08 | T | C | 0.0189385016678864 | 0.00346414883261138 | rs2885208 |
| 11 | 1,24E+08 | T | C | -0.0248216461781285 | 0.00444752664005169 | rs17128423 |
| 11 | 1,34E+08 | T | C | 0.0174304732843931 | 0.00285324493114963 | rs329672 |
| 12 | 15532891 | T | C | 0.0213685992144936 | 0.00367473761212272 | rs55754731 |
| 12 | 49385699 | T | C | 0.0475426411283091 | 0.00822679375814312 | rs146865992 |
| 12 | 49389320 | A | C | -0.0214634585236752 | 0.0028163572396897 | rs1054442 |
| 12 | 58292707 | A | G | -0.0195326852753319 | 0.00286277081567227 | rs1962047 |
| 12 | 79592680 | T | C | -0.0194808789980114 | 0.00282741349753431 | rs6539284 |
| 12 | 93176205 | A | T | 0.0171497415109487 | 0.00301189699876163 | rs17790352 |
| 12 | 1,24E+08 | A | G | 0.0178169423615842 | 0.00300657144137431 | rs1727307 |
| 13 | 55703218 | A | G | -0.0154125929728539 | 0.0028243710780381 | rs9569206 |
| 13 | 58635985 | T | G | -0.0176023820811371 | 0.00305649975362686 | rs520924 |
| 13 | 97847992 | A | G | 0.0334267803172284 | 0.00609643996301812 | rs9516855 |
| 13 | 1,07E+08 | T | C | 0.020104460465321 | 0.003648060327585 | rs17514375 |
| 13 | 1,07E+08 | C | G | -0.0257854741855271 | 0.0031270281573523 | rs2478286 |
| 14 | 27028189 | A | G | -0.01744622006403 | 0.00294004382609201 | rs178165 |
| 14 | 29600359 | T | C | 0.0261409472040691 | 0.00398915721105893 | rs176217 |
| 14 | 30074687 | T | C | -0.0167493611562435 | 0.00280746918475418 | rs971681 |
| 14 | 33292743 | A | C | -0.0205367452476897 | 0.00276626417668234 | rs2239647 |
| 14 | 37002135 | T | C | -0.0175077841002631 | 0.00282383614520372 | rs11622558 |
| 14 | 41134168 | A | G | 0.0197975304558593 | 0.00282337855902158 | rs35760956 |
| 14 | 69716957 | T | C | 0.0169111107971034 | 0.00302470234253325 | rs17106817 |
| 14 | 73463479 | A | G | 0.0160767364546037 | 0.0028057131683427 | rs1007934 |
| 14 | 98546911 | T | C | 0.0190329789390073 | 0.00317692854932521 | rs17698580 |
| 14 | 1,04E+08 | T | C | -0.0219741031986099 | 0.00285897777759691 | rs2071407 |
| 15 | 40722781 | T | G | 0.0220323719000254 | 0.00385720796569072 | rs11634187 |
| 15 | 41356439 | A | G | -0.0568445045045258 | 0.0101635087617604 | rs75322822 |
| 15 | 46640956 | T | C | -0.0154083820489327 | 0.00279390426997873 | rs55881236 |
| 15 | 51817198 | T | G | 0.0606344986951797 | 0.0090838200292404 | rs7172979 |
| 15 | 65738080 | T | C | -0.0343573247820526 | 0.00564808890055115 | rs72739469 |
| 15 | 82521770 | A | G | 0.0170306124889385 | 0.00274864630228188 | rs8025964 |
| 15 | 88430934 | T | C | 0.0176325609160892 | 0.00289628135941018 | rs1369429 |
| 16 | 5811367 | T | C | 0.0169362341701373 | 0.00304170872308501 | rs11076962 |
| 16 | 7666088 | T | G | 0.0177353824812975 | 0.00277158657310478 | rs11646221 |
| 16 | 10101152 | A | C | 0.0271302698457729 | 0.00458979357905142 | rs72774059 |
| 16 | 12197441 | A | C | -0.019751049026646 | 0.00313111113294958 | rs2457192 |
| 16 | 13026941 | A | G | 0.0196160193006761 | 0.00314863873205073 | rs62029752 |
| 16 | 13156649 | A | G | 0.0193456449981709 | 0.00354510628516967 | rs276626 |
| 16 | 13630944 | A | G | 0.0228700024783118 | 0.00381166707971864 | rs4781499 |
| 16 | 13914784 | A | C | -0.0202387238068202 | 0.00349546179737827 | rs9788857 |
| 16 | 24766841 | T | C | -0.0210951453358186 | 0.0029619692973629 | rs34172651 |
| 16 | 28825605 | A | G | -0.0286784887809439 | 0.00279926684050209 | rs2008514 |
| 16 | 51577196 | T | C | -0.0197491157569477 | 0.00304394509200797 | rs2647995 |
| 16 | 53424943 | T | C | -0.0192759864179111 | 0.00286802357058639 | rs1990634 |
| 16 | 62075138 | A | G | 0.0160541191107679 | 0.00274241870699827 | rs12446238 |
| 16 | 68297228 | A | G | -0.0235019481325088 | 0.00426223216041145 | rs9888986 |
| 16 | 70756066 | T | C | 0.0152421098269666 | 0.00278598242130627 | rs7196032 |
| 16 | 71870700 | A | G | 0.0189239778933522 | 0.0031456080274854 | rs8051038 |
| 17 | 34951204 | A | G | 0.0175136933174277 | 0.00276459247315354 | rs2285640 |
| 17 | 42919009 | A | G | -0.0177187238934688 | 0.00324994935683581 | rs4793161 |
| 17 | 44819595 | T | G | -0.0201149356410057 | 0.00356647795053292 | rs17698176 |
| 17 | 47090785 | T | C | 0.0165478124348439 | 0.00295972320422892 | rs11079849 |
| 17 | 50585898 | T | G | -0.0192497233184071 | 0.00309979441520244 | rs16951547 |
| 17 | 56999427 | A | G | -0.0208823048430228 | 0.00283380442977647 | rs66954617 |
| 18 | 50052084 | A | C | 0.0559740276901206 | 0.00874594182658135 | rs71367283 |
| 18 | 50831176 | A | G | -0.022748050061836 | 0.00273743081369868 | rs6508220 |
| 19 | 4474725 | A | C | 0.042223404623821 | 0.00758050352312765 | rs76608582 |
| 19 | 12530177 | A | G | 0.0255982205662394 | 0.00426139846283326 | rs17002025 |
| 19 | 13113641 | T | C | 0.0164085648684515 | 0.00275866927848882 | rs10411958 |
| 19 | 18257750 | T | C | 0.0169958646136373 | 0.00274481017662101 | rs2072490 |
| 19 | 31929180 | T | C | -0.0191750040250758 | 0.00282026827843445 | rs7248006 |
| 19 | 39583980 | T | C | 0.041306588425032 | 0.00746819533990815 | rs144026674 |
| 19 | 47548678 | A | G | 0.0160710675286269 | 0.00289204022469443 | rs889169 |
| 19 | 59035589 | T | C | 0.0184739662033539 | 0.0030662184569882 | rs113742427 |
| 20 | 34219990 | A | C | -0.0228754796786845 | 0.00404947418634882 | rs78084033 |
| 20 | 47541517 | A | G | 0.0251054480871655 | 0.00297598957884845 | rs6019535 |
| 21 | 40516070 | A | G | 0.0203464724967938 | 0.00296293468716963 | rs2836921 |
| 22 | 31304201 | A | G | 0.0159100222255461 | 0.00291873458549736 | rs5753383 |
| 22 | 38144618 | T | C | 0.0152173451530449 | 0.00277486235467631 | rs5756805 |
| 22 | 39840828 | A | C | 0.0228910533156975 | 0.00312676592210047 | rs5750830 |
| 22 | 39976186 | T | C | -0.0178385937685344 | 0.00282211576784281 | rs5750853 |
| 22 | 41461836 | A | G | 0.0159990863375022 | 0.00287494812893121 | rs4821995 |
| 22 | 41992169 | A | G | 0.0353632552974441 | 0.00497723508760648 | rs62236533 |

| **Table e-3.** Associations of physical and cognitive fitness in late adolescence with the future risk of Parkinson's disease (PD) defined as main diagnosis only | | | |
| --- | --- | --- | --- |
| **Characteristics** | **No. of PD** | **HR^a^ (95% CI)** | **HR^b^ (95% CI)** |
|  |  |  |  |
| **Physical fitness (Watts)** |  |  |  |
| <232 | 569 | 1.00 | 1.00 |
| 233-289 | 291 | 1.09 (0.94-1.27) | 1.11 (0.95-1.30) |
| >290 | 124 | 1.09 (0.88-1.35) | 1.16 (0.92-1.47) |
| **Body mass index (kg/m^2^)** |  |  |  |
| Underweight (<18.5) | 118 | 1.12 (0.92-1.36) | 1.11 (0.91-1.37) |
| Normal (18.5-24.99) | 762 | 1.00 | 1.00 |
| Overweight (>25) | 84 | 1.11 (0.89-1.39) | 1.16 (0.92-1.47) |
| **Resting heart rate (beats per minute)** |  |  |  |
| Slow (<60) | 109 | 1.06 (0.87-1.30) | 1.02 (0.83-1.25) |
| Normal (60-100) | 766 | 1.00 | 1.00 |
| Fast (>100) | 41 | **1.45 (1.06-1.98)** | **1.52 (1.11-2.09)** |
| **Blood pressure (mmHg)** |  |  |  |
| Normal (<120/<80) | 169 | 1.00 | 1.00 |
| Elevated (120-129/<80) | 288 | 1.07 (0.89-1.29) | 1.07 (0.88-1.30) |
| Hypertension (≥130/≥80) | 507 | 0.95 (0.80-1.13) | 0.92 (0.77-1.10) |
| **IQ (stanine)** |  |  |  |
| 1-3 | 158 | 1.00 | 1.00 |
| 4-6 | 498 | 1.18 (0.99-1.42) | 1.18 (0.98-1.42) |
| 7-9 | 307 | **1.47 (1.21-1.78)** | **1.50 (1.22-1.85)** |
| **Stress resilience (stanine)** |  |  |  |
| 1-3 | 195 | 1.00 | 1.00 |
| 4-6 | 522 | 0.93 (0.79-1.10) | 0.86 (0.72-1.02) |
| 7-9 | 240 | 1.07 (0.88-1.29) | 0.93 (0.75-1.14) |
| ^a^HR: hazard ratio; adjusted for attained age, calendar period of conscription, and parental socioeconomic status  ^b^HR: hazard ratio; adjusted for attained age, calendar period of conscription, parental socioeconomic status, physical fitness, body mass index, resting heart rate, blood pressure, IQ, and stress resilience | | | |

| **Table e-4.** Associations of resting heart rate and IQ in late adolescence with the future risk of Parkinson's disease defined as main diagnosis only, after additional adjustment for adult education and cardiovascular disease | |
| --- | --- |
| **Characteristics** | **HR (95% CI)** |
|  |  |
| **Resting heart rate (beats per minute)** |  |
| Slow (<60) | 1.01 (0.82-1.24) |
| Normal (60-100) | 1.00 |
| Fast (>100) | **1.53 (1.11-2.10)** |
| **IQ (stanine)** |  |
| 1-3 | 1.00 |
| 4-6 | 1.15 (0.95-1.40) |
| 7-9 | **1.42 (1.14-1.77)** |
| HR: hazard ratio; adjusted for attained age, calendar period of conscription, parental socioeconomic status, physical fitness, body mass index, resting heart rate, blood pressure, IQ, stress resilience, and adult education and cardiovascular diseases | |

| **Table e-5.** Effects of PD on resting heart rate and intelligence: a Mendelian randomization analysis | | |
| --- | --- | --- |
|  | Β (95% CI) | P value |
| **Resting heart rate** |  |  |
| IVW | 0.04 (-0.13, 0.21) | 0.66 |
| Weighted Median | 0.14 (-0.04, 0.32) | 0.14 |
| MR-Egger | 0.30 (-0.07, 0.67) | 0.11 |
|  |  |  |
| **Intelligence** |  |  |
| IVW | 0.01 (-0.01, 0.02) | 0.47 |
| Weighted Median | 0.01 (-0.01, 0.02) | 0.48 |
| MR-Egger | 0.01 (-0.02, 0.04) | 0.46 |
|  |  |  |
| IVW: inverse variance weighted; MR: Mendelian randomization | | |
