# Supplementary figures and images for: Heart rate, intelligence in adolescence, and Parkinson’s disease later in life
Source: Eur J Epidemiol. 2021 Mar 6;36(10):1055–64. doi: 10.1007/s10654-021-00730-y (PMC8542538; doi:10.1007/s10654-021-00730-y)

# MR Test

- Inverse variance weighted
- MR Egger
- Weighted median

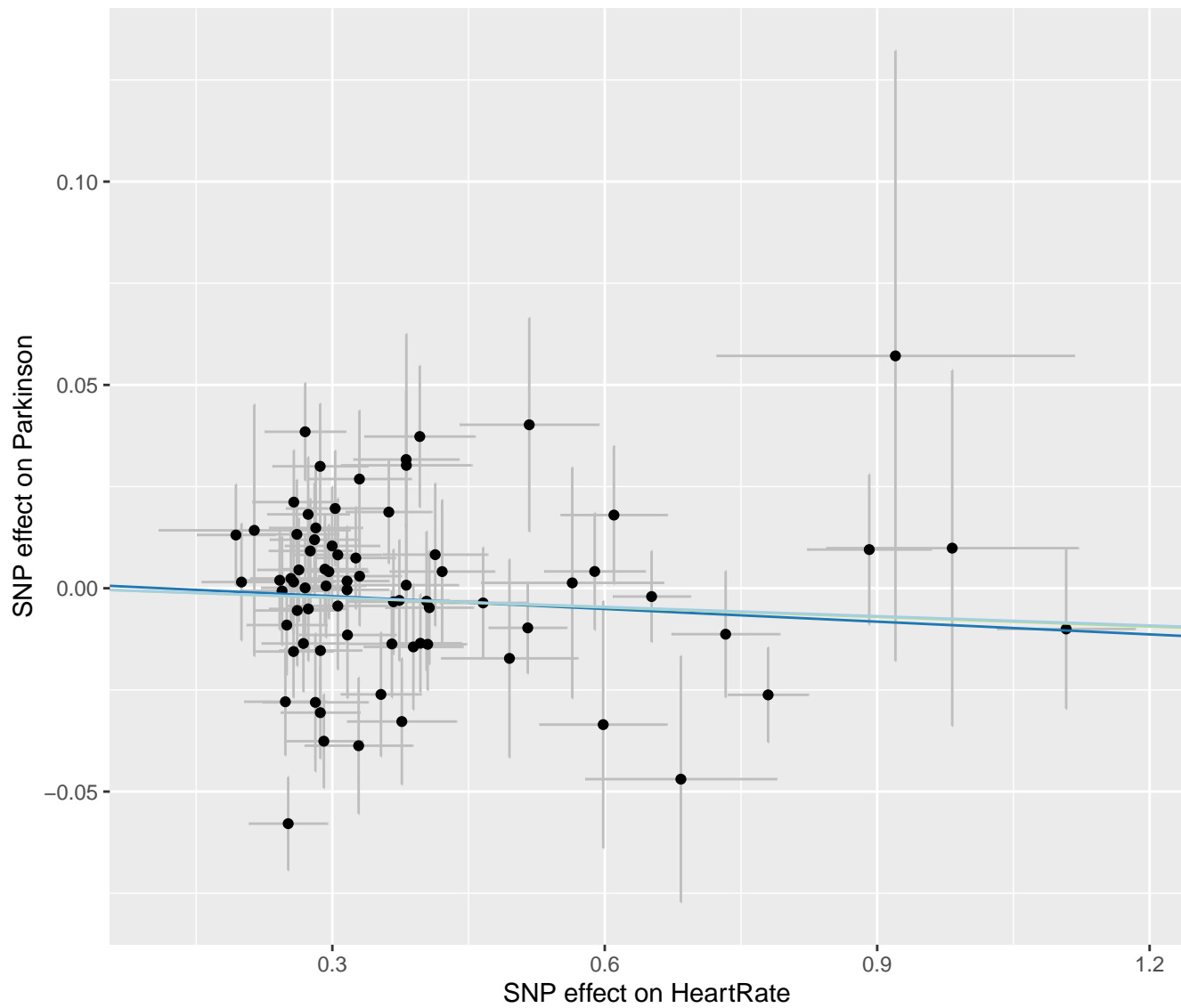

Supplement: Supplementary file 2 — Supplementary file2 (PDF 8 KB) [file 10654_2021_730_MOESM2_ESM.pdf]

# MR Test

- Inverse variance weighted
- MR Egger
- Weighted median

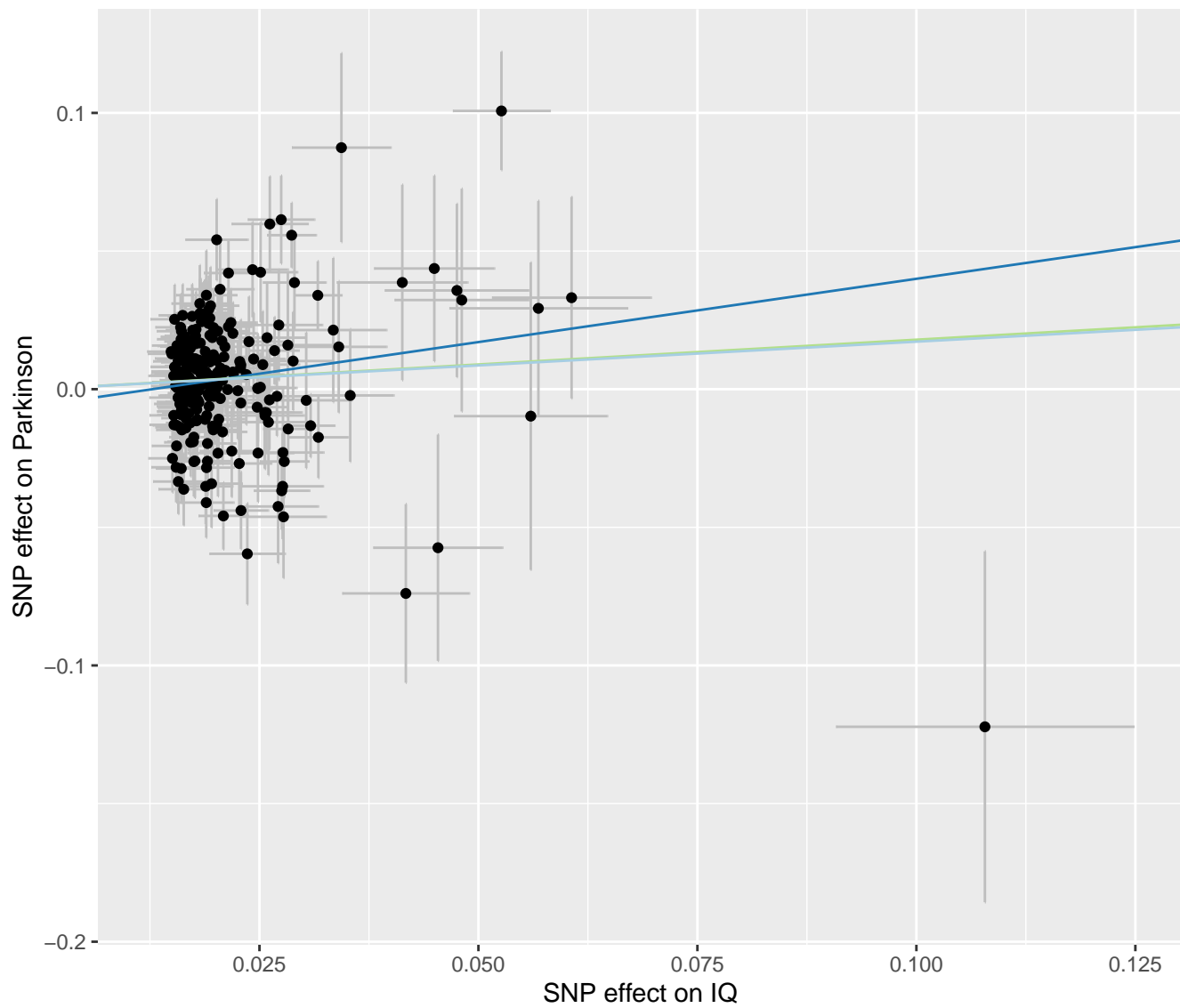

Supplement: Supplementary file 3 — Supplementary file3 (PDF 15 KB) [file 10654_2021_730_MOESM3_ESM.pdf]

# MR Test

- Inverse variance weighted
- MR Egger
- Weighted median

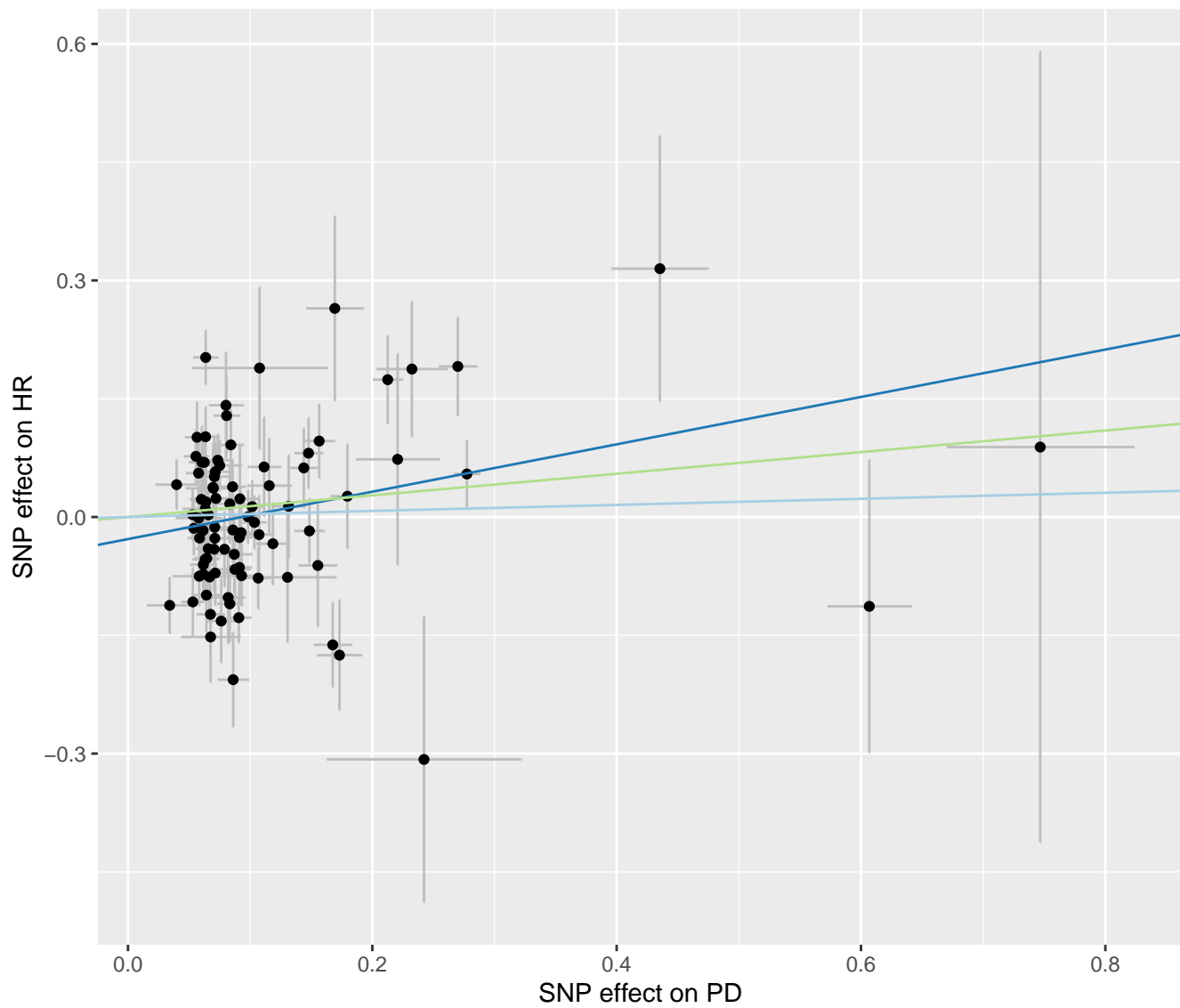

Supplement: Supplementary file 4 — Supplementary file4 (PDF 9 KB) [file 10654_2021_730_MOESM4_ESM.pdf]

# MR Test

- Inverse variance weighted
- MR Egger
- Weighted median

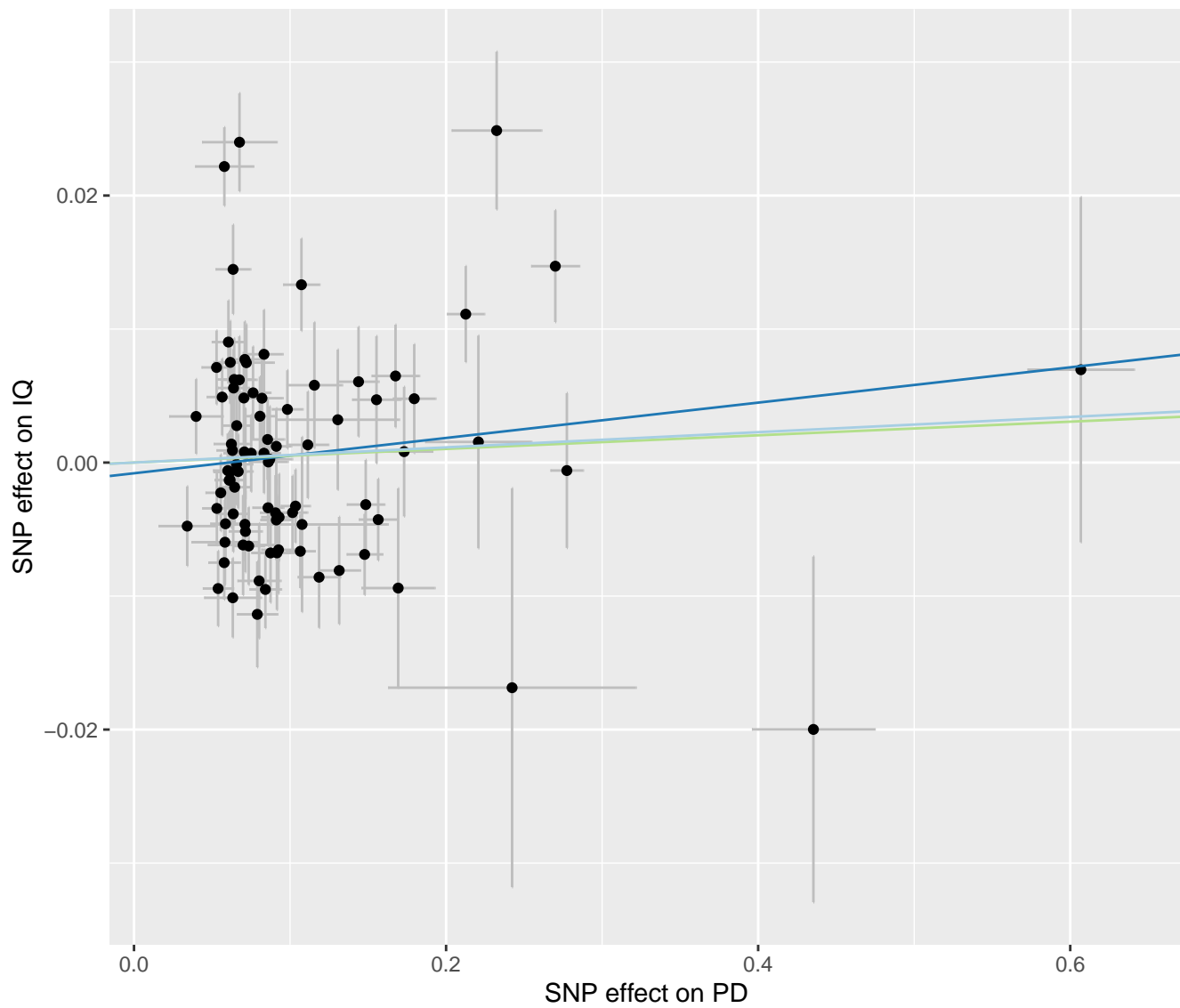

Supplement: Supplementary file 5 — Supplementary file5 (PDF 9 KB) [file 10654_2021_730_MOESM5_ESM.pdf]

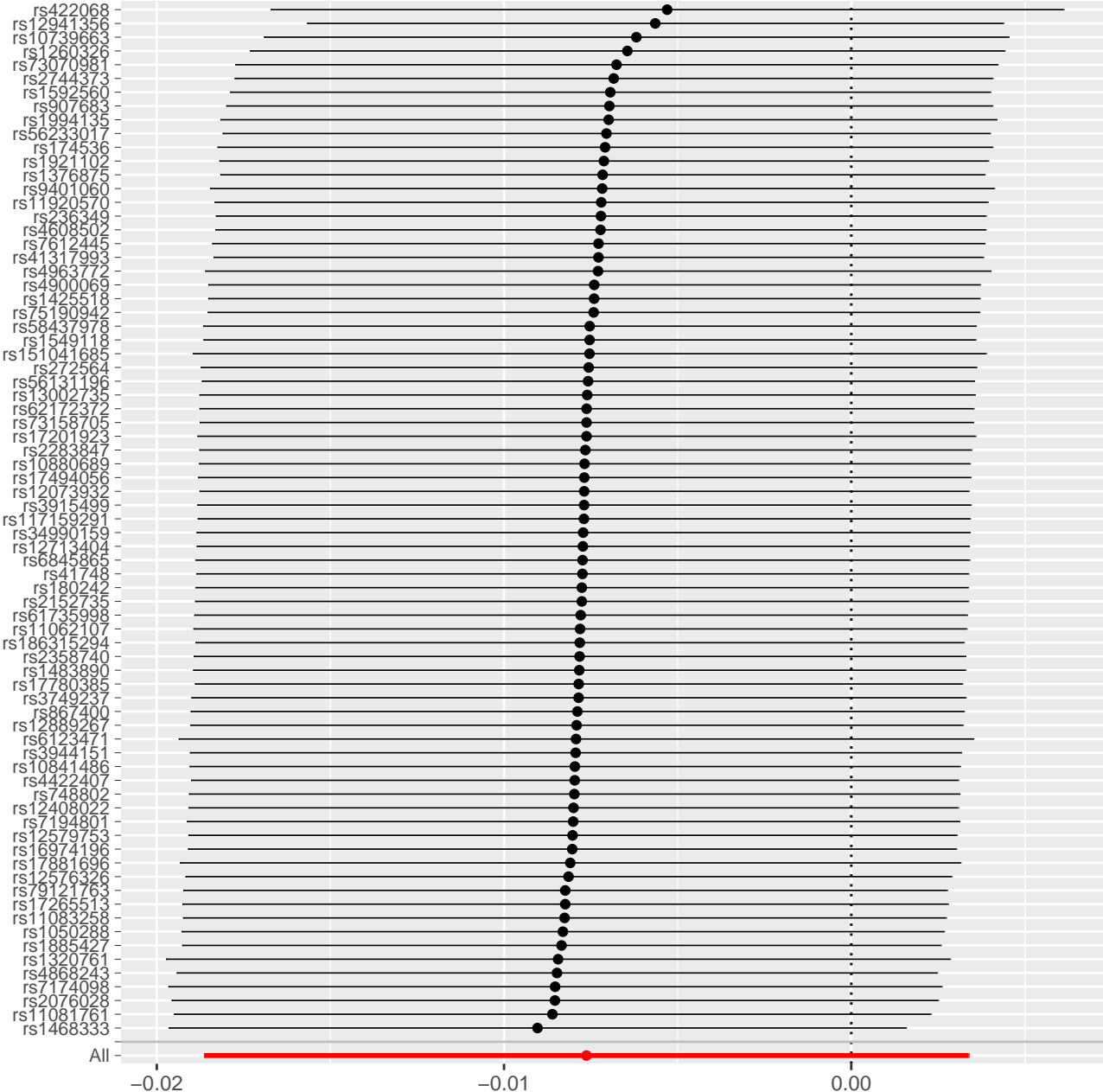

MR leave-one-out sensitivity analysis for 'HeartRate' on 'Parkinson'

Supplement: Supplementary file 6 — Supplementary file6 (PDF 8 KB) [file 10654_2021_730_MOESM6_ESM.pdf]

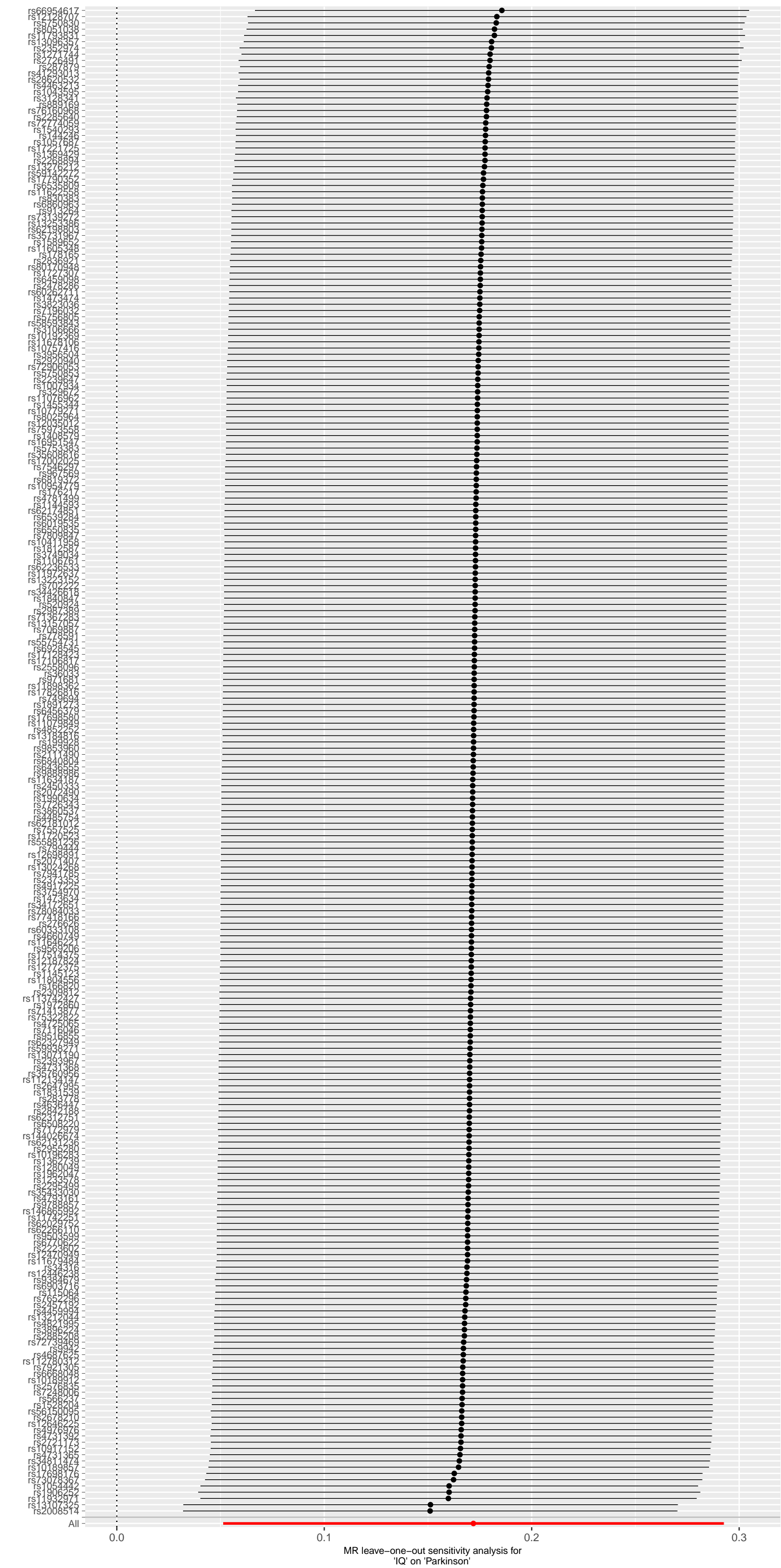

Supplement: Supplementary file 7 — Supplementary file7 (PDF 17 KB) [file 10654_2021_730_MOESM7_ESM.pdf]

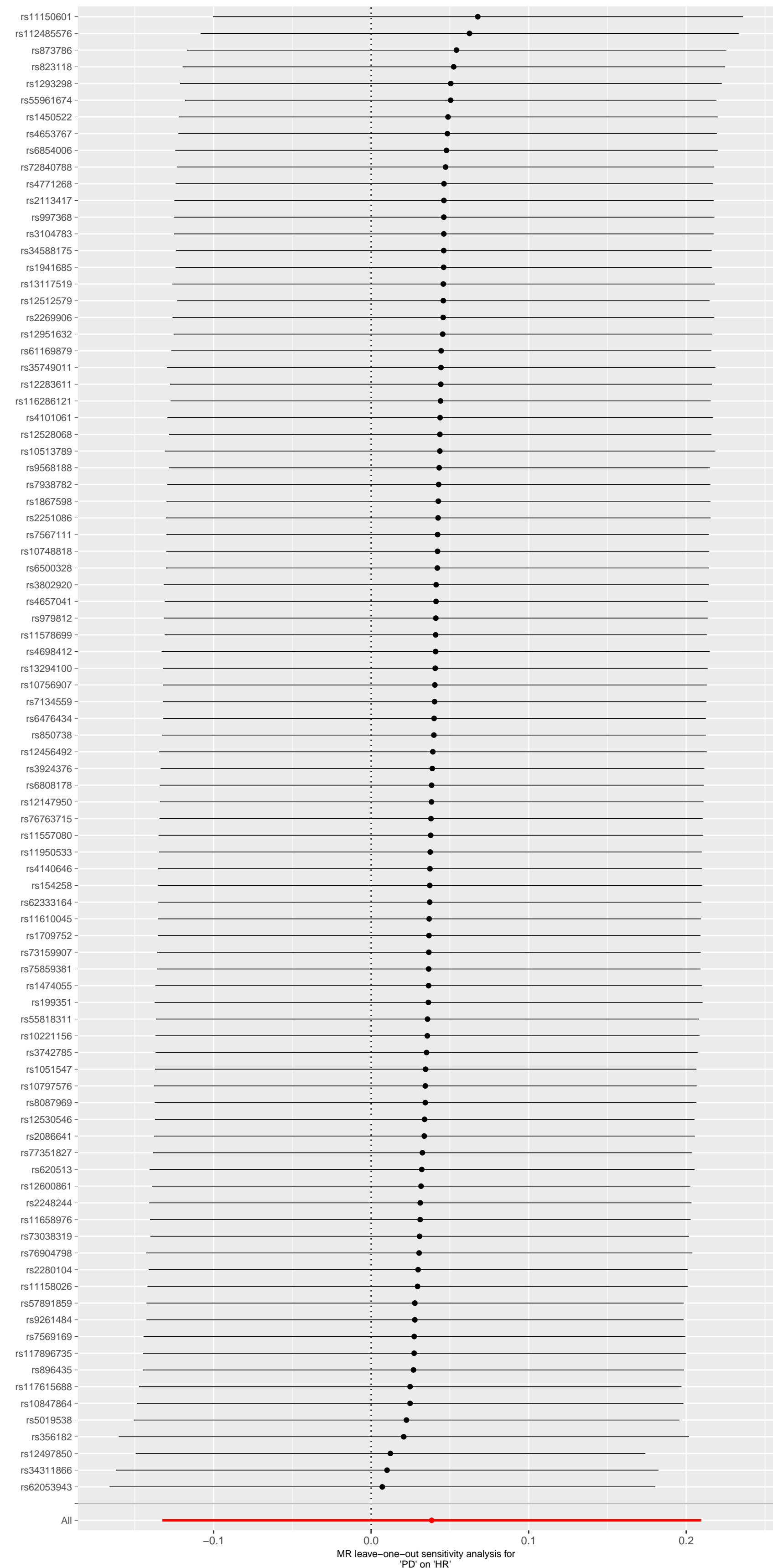

Supplement: Supplementary file 8 — Supplementary file8 (PDF 9 KB) [file 10654_2021_730_MOESM8_ESM.pdf]

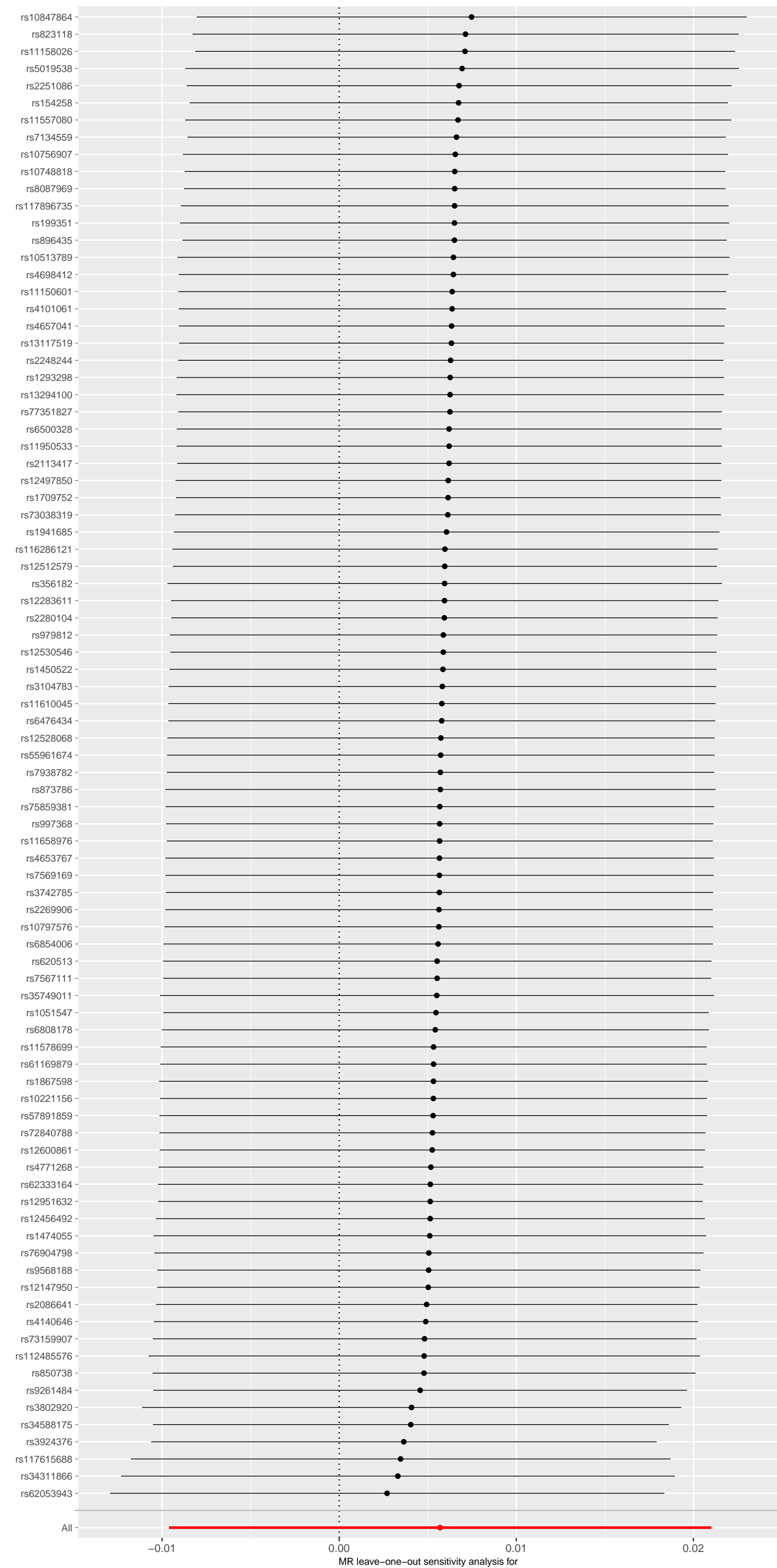

Supplement: Supplementary file 9 — Supplementary file9 (PDF 9 KB) [file 10654_2021_730_MOESM9_ESM.pdf]

# MR Method

- Inverse variance weighted
- MR Egger

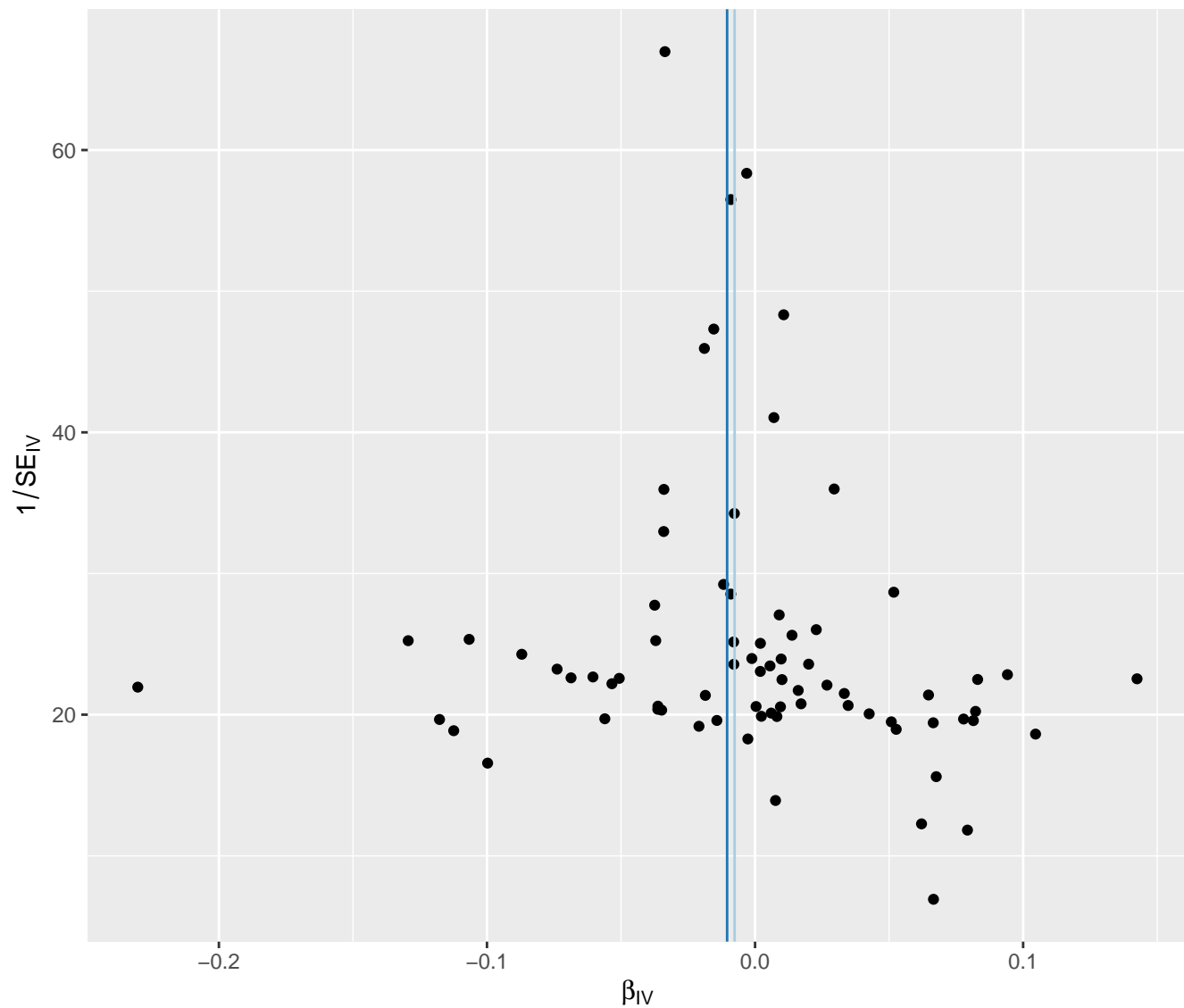

Supplement: Supplementary file 10 — Supplementary file10 (PDF 6 KB) [file 10654_2021_730_MOESM10_ESM.pdf]

# MR Method

- Inverse variance weighted
- MR Egger

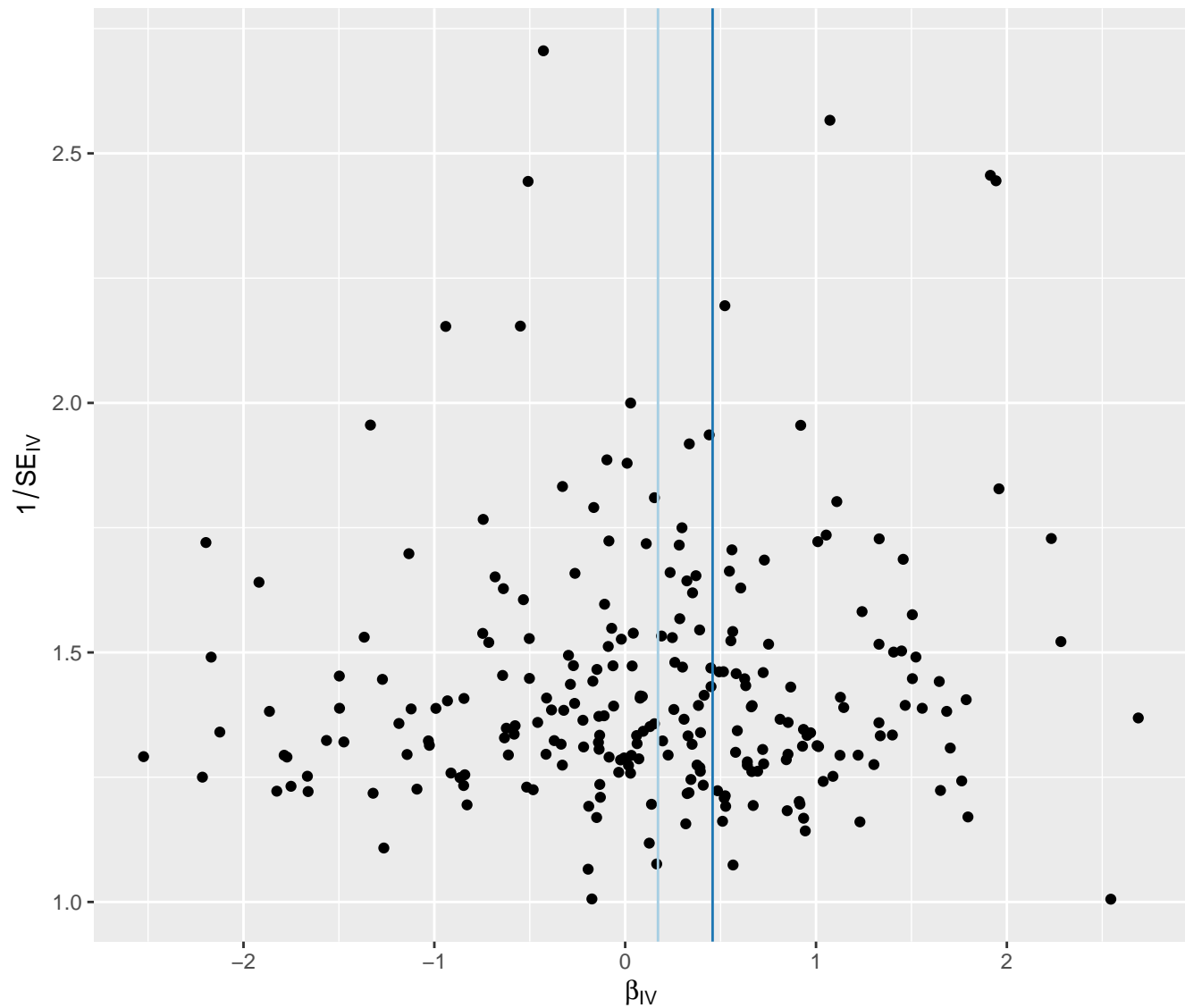

Supplement: Supplementary file 11 — Supplementary file11 (PDF 7 KB) [file 10654_2021_730_MOESM11_ESM.pdf]

# MR Method

- Inverse variance weighted
- MR Egger

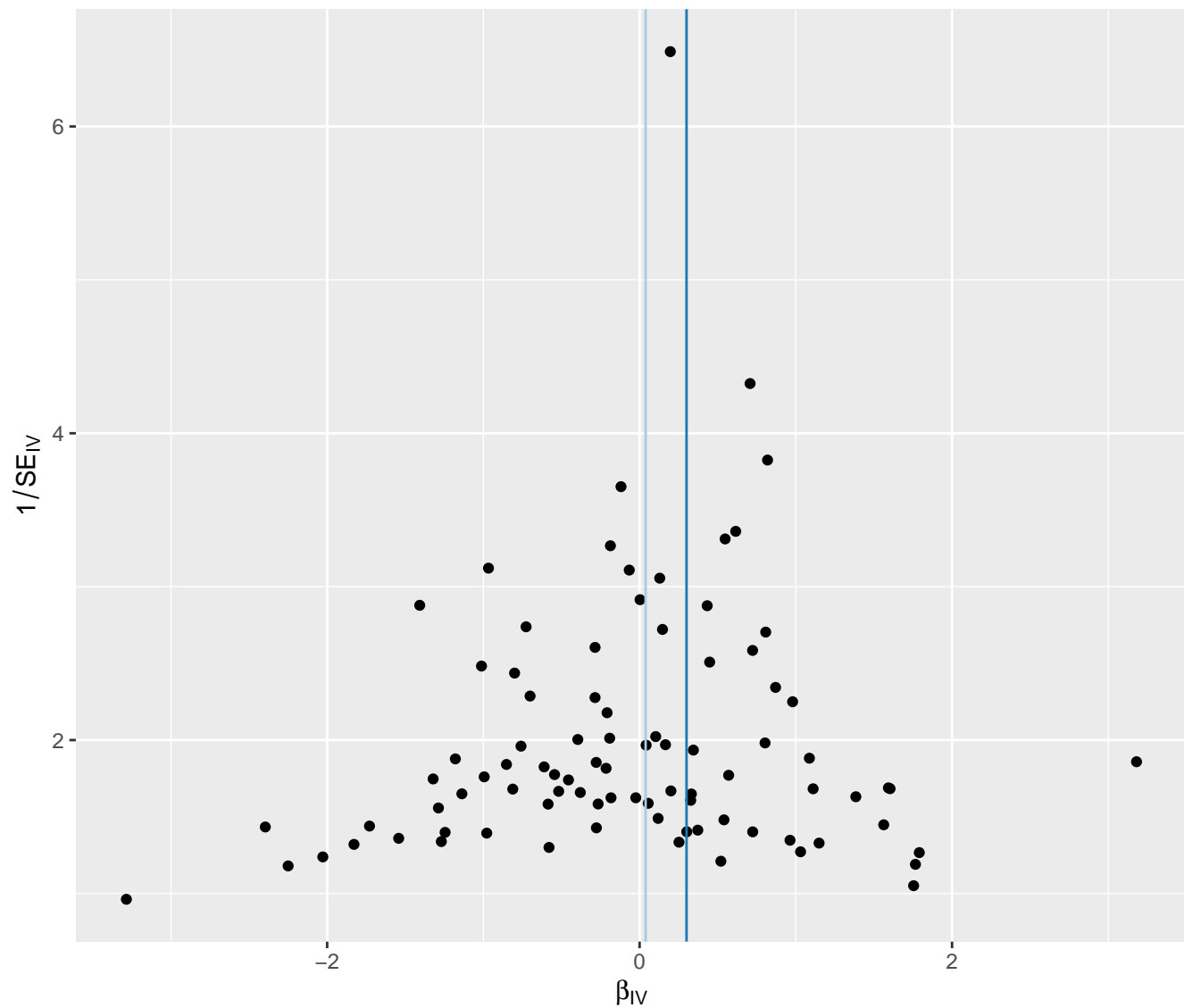

Supplement: Supplementary file 12 — Supplementary file12 (PDF 6 KB) [file 10654_2021_730_MOESM12_ESM.pdf]

# MR Method

- Inverse variance weighted
- MR Egger

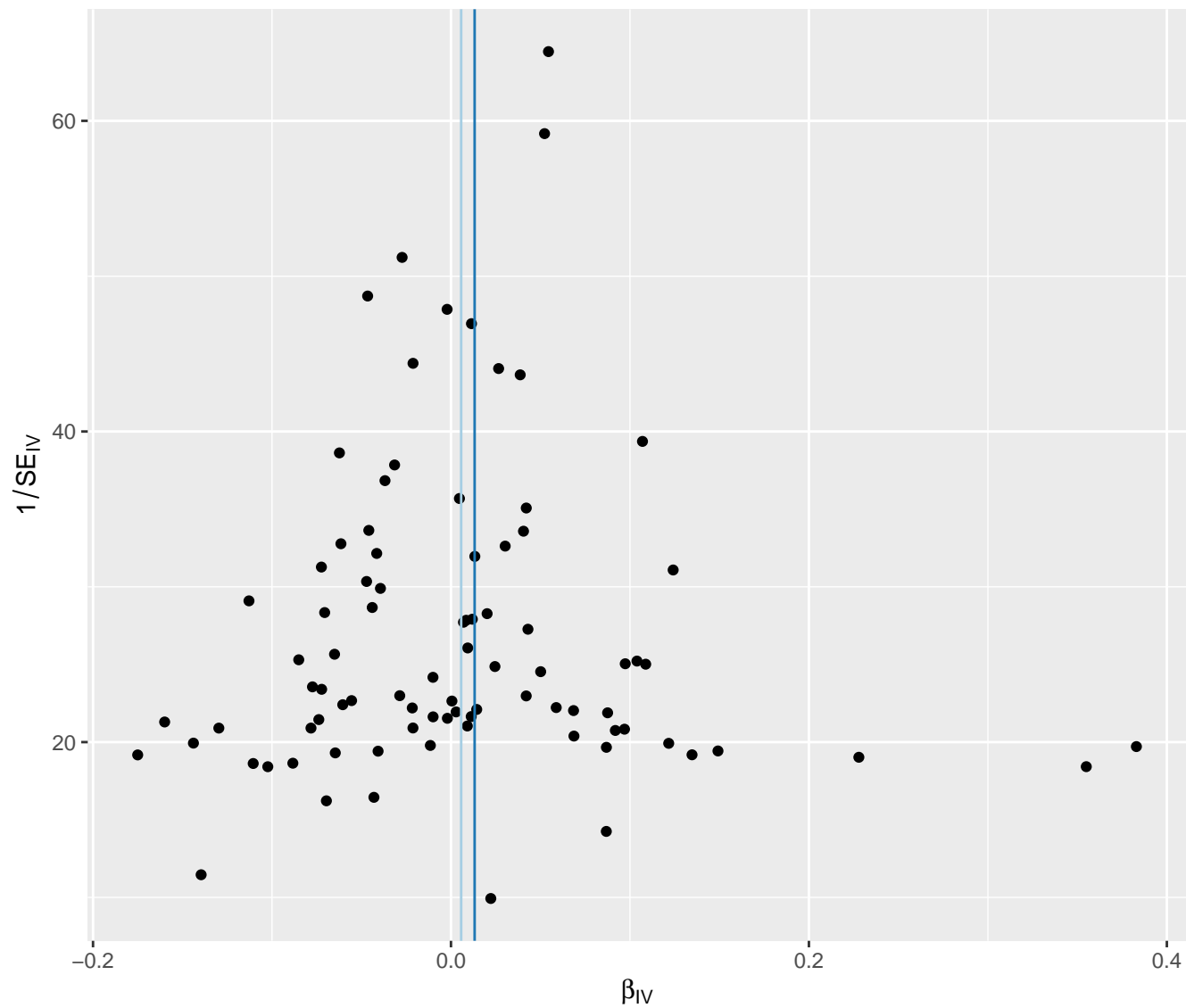

Supplement: Supplementary file 13 — Supplementary file13 (PDF 6 KB) [file 10654_2021_730_MOESM13_ESM.pdf]
